# Using Science and Technology to Unveil The Hidden Delicacy *Terfezia arenaria*, a Desert Truffle

**DOI:** 10.3390/foods12193527

**Published:** 2023-09-22

**Authors:** Inês Ferreira, Teresa Dias, Abdul M. Mouazen, Cristina Cruz

**Affiliations:** 1 cE3c—Centre for Ecology, Evolution and Environmental Changes & CHANGE, Global Change and Sustainability Institute, Faculdade de Ciências, Universidade de Lisboa, Campo Grande, Bloco C2, 1749-016 Lisboa, Portugal; iiferreira@fc.ul.pt (I.F.); ccruz@fc.ul.pt (C.C.); 2 Department of Environment, Faculty of Bioscience Engineering, Ghent University, Coupure Links 653, 9000 Ghent, Belgium; abdul.mouazen@ugent.be

**Keywords:** desert truffles, electronic nose, mushrooms and truffles, nutritional composition, plant-based meat, volatile organic compounds

## Abstract

*Terfezia arenaria* is a desert truffle native to the Mediterranean Basin region, highly appreciated for its nutritional and aromatic properties. Despite the increasing interest in this desert truffle, *T. arenaria* is not listed as an edible truffle authorized for trade in the European Union. Therefore, our objective was to showcase *T. arenaria*’s nutritional and chemical composition and volatile profile. The nutritional analysis showed that *T. arenaria* is a good source of carbohydrates (67%), proteins (14%), and dietary fibre (10%), resulting in a Nutri-Score A. The truffle’s volatile profile was dominated by eight-carbon volatile compounds, with 1-octen-3-ol being the most abundant (64%), and 29 compounds were reported for the first time for *T. arenaria*. *T. arenaria*’s nutritional and chemical compositions were similar to those of four commercial mushroom and truffle species, while the aromatic profile was not. An electronic nose corroborated that *T. arenaria*‘s aromatic profile differs from that of the other four tested mushroom and truffle species. Our data showed that *T. arenaria* is a valuable food resource with a unique aroma and an analogous composition to meat, which makes it an ideal source for plant-based meat products. Our findings could help promote a sustainable future exploitation of *T. arenaria* and ensure the quality and authenticity of this delicacy.

## 1. Introduction

Food production currently faces many challenges [1]. One of these challenges is climate change, which causes severe health, economic and environmental problems [2]. Current political actions, such as the United Nation’s Sustainable Development Goals (e.g., SDG 12 Sustainable consumption and production, SDG13 Climate action), aim to mitigate climate change by shifting production and consumption patterns, and the production of mycorrhizal sporocarps (i.e., the fruiting body of macrofungi usually known as mushroom or truffle) is no exception [2,3]. With a key role in ecosystems [4], ectomycorrhizal fungi provide services and goods essential to maintain soil quality, ecosystem functions and food (some species) [2].

The global trade of mushrooms and truffles has grown significantly in the last two decades [5,6]; in 2000, the global production of mushrooms and truffles was 8.78 million tons, and by 2021, this number had grown to 44.20 million tons. This represents a growth of over four times. The average producer price of mushrooms and truffles also increased during this period, by 1.5 times. However, a wider investment in mycorrhizal mushrooms and truffles as a food source, with the associated health, environmental and economic benefits [7,8], is still hampered by insufficient science to showcase its benefits as a food source, and technology to boost its sustainable production and ensure its reliable identification. In agreement, although the production of mycorrhizal sporocarps has long had a high economic, cultural and environmental impact in the Mediterranean Basin region [2], the list of the edible sporocarps authorized for trade in 27 European countries only includes 12 species.

Furthermore, insufficient data make it challenging to provide accurate information on mycorrhizal mushrooms and truffles production and market prices. Taking the desert truffles (i.e., a family of truffles endemic to arid and semiarid areas of the Mediterranean Basin Region, North Africa and the Middle East, which includes several genera, namely *Terfezia*, *Tirmania* and *Mattirolomyces*) as an example, they are among the wild mushrooms and truffles with higher selling prices [9,10]. Due to strong cultural traditions [11], desert truffles have been part of the Mediterranean, North African and Middle Eastern cultures for centuries [12], and are widely consumed in these regions [12,13]. As most of the world’s trade of desert truffles occurs in North African countries [14], and most of this regional trade is not official, there is a lack of official data in these countries [8]. Therefore, the world’s production and annual market of desert truffles is still unknown [14]. Nevertheless, desert truffle plantation yields can reach approximately 350 kg ha^−1^, representing an expected average income of 7000 EUR ha^−1^ [14]. Besides the economic revenue, desert truffles also constitute an important nourishment source in North African and Arabic countries, being frequently used as a meat substitute [11,15] or as a powder to increment the nutritional quality of bread and biscuits [16,17]. Despite its great potential as a food source, only one edible desert truffle species is widely traded: *Terfezia claveryi.* All other potentially edible desert truffle species are being ignored. Therefore, a wider investment in desert truffles (and other mycorrhizal sporocarps) as a food source, with the associated health, environmental and economic benefits, is being hampered by insufficient science to showcase its benefits as a food source, and technology to boost its sustainable production and ensure its reliable identification.

*Terfezia areanaria* (Moris) Trappe is another desert truffle that forms a seasonal edible truffle with important ecological and socio-economic relevance [18]. Some studies have shown that this species, like other desert truffles, is rich in carbohydrates, proteins, and dietary fibre, making it a suitable addition to a balanced diet [19,20,21,22,23,24]. *T. arenaria* has also been reported to have important biological activities, such as antioxidant, antimicrobial and antitumoral [23,25,26,27]. Despite the increasing interest in exploring the nutritional and chemical composition of desert truffles [19,20,21,22], *T. arenaria*’s consumption and trade are still limited to small regions where this species is native. Therefore, one of our objectives was to showcase *T. arenaria*’s nutritional value by comparing it with other edible mushrooms and truffles, namely species that are well-known by the consumer and are widely available in the market.

Furthermore, ensuring food security and authentication are vital strategies for the sustainable exploitation of this native resource, preserving its long-term viability and conservation. Desert truffles are hypogeous (i.e., mushroom formation occurs belowground), which makes them difficult to detect [28]. However, as *T. arenaria* (and other desert truffle species) are mycorrhizal fungi that are associated with a host plant (most frequently from the Cistaceae family [29]; *T. arenaria* associates with the annual plant species *Tuberaria guttata* [30]), and screening the potential host plants is part of the detection method. Once their potential location is detected, the traditional method of harvesting desert truffles involves a pointed stick to carefully probe the soil [13]. This potentially destructive technique is time-honoured and traditionally passed from generation to generation, predominantly in Mediterranean regions [15,31]. However, harvesting desert truffles is a difficult process practiced by specialists [11], which are becoming fewer and fewer among the new generations due to the abandonment of rural areas and traditional forestry [2,14]. Desert truffles’ (and wild mushrooms in general) incorrect harvesting, including excessive harvesting, can lead to the destruction of fungal structures, making future productivity unfeasible [32]. In the case of incorrect species identification, it can cause poisoning, leading to a feeling of insecurity (mycophobia) among consumers [3,33]. Like the mushrooms and truffles commonly found in supermarkets, it becomes crucial to establish comprehensive knowledge regarding the safe consumption of desert truffles and other wild mushrooms and truffles. Altogether, developing and implementing guidelines that ensure food safety becomes especially significant for the mushroom trade [33]. Only by prioritizing the development of sustainable harvesting techniques and tools to assess quality and authenticity can we establish a fair value chain for these endogenous products. These steps are essential to meet consumer’s health and nutritional needs while safeguarding the resource and promoting equitable practices in its utilization.

So far, *T. arenaria* and other desert truffle species identification has relied on traditional knowledge and morphological identification by experts. However, besides its nutritional value and potential health benefits, *T. arenaria* has a unique bouquet of volatile organic compounds (VOCs; includes alcohols, aldehydes, ketones, and sulphur compounds [34,35,36]), which is perceived by humans as a subtle, sweet and agreeable flavour [11,13] and contributes to promote its quality and gastronomic value [15,31]. Therefore, we consider that *T. arenaria*’s VOCs bouquet could be explored to develop a robust and efficient analysis method to certify the quality and authenticity of this delicacy.

Studies on the VOCs present in desert truffle species are still scarce [35], and only one study included *T. arenaria* [34]. Currently, the most common identification and quantification methods for VOCs analysis is gas chromatography–mass spectrometry (GC–MS) [37], and it has been widely applied to truffles and desert truffles [35,38]. GC–MS is a powerful analytical technique with high sensitivity, easy metabolite identification, and has the possibility to couple with separation techniques [39]. However, it can be time consuming to prepare samples as it is a destructive analysis operated by highly qualified technicians and it is very expensive [39,40]. On the other hand, the use of electronic nose (e-nose) technology has gained attention in recent years for the identification and analysis of aroma profiles in mushrooms [41,42,43,44] and other food products [45,46]. This methodology has been frequently combined with GC–MS analysis, as a non-destructive and rapid approach to quality control and product authentication [37]. The e-nose was proven a successful methodology to distinguish between the volatile profile of several filamentous fungi species and/or strains, for health, environmental and food control applications [47]. In the case of mushrooms, most studies reported the volatile profile in relation with quality analysis in post-harvest processes [43,45,48,49,50,51]. Similarly to what was reported for filamentous fungi, this technology can also be applied for the identification and differentiation of mushroom and truffles species [41,52,53,54,55]. The use of e-noses in the food industry is widespread, with applications in meat, dairy products, aquatic products, cereals, fruits, and vegetables. Advantages of the e-nose include their rapid response, low cost and a relatively simple operating process [56]. Therefore, given that *T. arenaria* has a unique bouquet of volatile organic compounds, we tested if the e-nose was capable of distinguishing *T. arenaria* from other edible mushroom and truffle species, and therefore guarantee this desert truffle’s authenticity. For that, we used the e-nose Cyranose-320 to analyse *T. arenaria*’s volatile profile, applying two pre-analysis incubation temperatures to understand if the temperature could affect VOCs emissions and compromise the e-nose’s identification efficiency: (a) *T. arenaria* samples incubated for one hour at 40 °C (40 °C); and (b) *T. arenaria* samples incubated for one hour at room temperature (RT). In the identification process, four commercial edible species (*Agaricus bisporus*, *Lentinula edodes*, *Pleurotus ostreatus* and *Tuber melanosporum*) were also tested to confirm the ability of Cyranose-320 to distinguish *T. arenaria* from other edible species.

Our review on *T. arenaria*’s nutritional and health value, and proposal for the first steps in developing a non-destructive and rapid identification method for early detection of *Terfezia* truffles, their growth stages, and quality are crucial for sustainable resource exploitation. This innovation could promote our understanding and management of desert truffle populations, ensuring their preservation and responsible production and use in the long term.

## 2. Materials and Methods

### 2.1. Terfezia arenaria Samples

Desert truffles naturally fruit in the spring from February to May. For three weeks of the 2019 spring season, sixty-three *T. arenaria* truffles were harvested in Alentejo (south of Portugal) (see Figure S1). The samples were collected in four sampling sites—S1, S2, S3 and S4—with an area of 200 m^2^ each; the sampling sites were separated by 1 to 2 km distance. In all the sampling sites, the Cistaceae host plants were abundant (*Tuberaria guttata*), but the forest-dominant species differed: *Quercus suber* in sites 1 and 2, *Pinus pinea* in site 3 or mixed in site 4 (Figure S1). Specimens were freed from substrate debris at the site and further cleaned in the laboratory and used to analyse *T. arenaria*’s nutritional and chemical composition and volatile profile (using 2 techniques). *T. arenaria* samples were (i) kept at −20 °C until molecular analysis; (ii) dried for nutritional and chemical composition analyses; and (iii) kept at 4 °C until volatile profile analysis in the first 48 h post-harvest.

The specimens were identified by molecular analysis.

For the nutritional and chemical analyses, we used three dry samples of *T. arenaria* truffles collected in three of the sampling sites (sites S1, S2, S3, S4) during the first week of April 2019. From each sampling site, one desert truffle with similar size and appearance was analysed.

For the volatiles profile analysis, we used three fresh samples of *T. arenaria* truffles collected in three of the sampling sites (sites S2, S3, S4) during the first week of April 2019. From each sampling site, one desert truffle with similar size and appearance was analysed.

To validate *T. arenaria*’s identification using the e-nose, we used a total of five mushroom and truffle species: *T. arenaria*, *A. bisporus*, *L. edodes*, *P. ostreatus* and *T. melanosporum.* Mature *T. arenaria* truffles were harvested in Alentejo (south of Portugal) as described in Section 2.1. *A. bisporus*, *L. edodes*, and *P. ostreatus* were purchased in a local supermarket, and *T. melanosporum* was purchased at Espora Gourmet, SL. All fresh mushrooms and truffles samples were kept at 4 °C until analysis in the first 48 h post-harvest.

### 2.2. Comparing T. arenaria’s Nutritional Value with That of Other Edible Mushrooms and Truffles, and Meat

To showcase its nutritional value, we collected and analysed *T. arenaria*’s samples for their nutritional and mineral composition. Furthermore, *T. arenaria*’s data was compared with that reported in the literature for other edible mushroom and truffle species (*A. bisporus*, *L. edodes*, *P. ostreatus* and *T. melanosporum*), and with meat (cow, pig and chicken). The criteria for selecting the other edible mushroom and truffle species and the types of meat were wide consumption and easy to buy and find in the supermarkets.

Twelve *T. arenaria* composite samples, three for each sampling site, were prepared. The samples were dried at 40 °C for 72 h to determine their moisture content, and the dry material was powdered in a porcelain mortar and kept in brand-new sealed polyethylene bags under dry conditions at room temperature until analysis. Using the AOAC procedures [57], the dry samples were analysed for their (i) crude protein content (applying the conversion factor of N × 4.38), which was estimated by the macro-Kjeldahl method, (ii) crude fat, which was determined by extracting a known weight of powdered sample with petroleum ether, using a Soxhlet apparatus, and (iii) ash concentration, which was determined by incineration at 600 ± 15 °C. A bomb calorimeter (Parr 6200 Isoperibol Calorimeter) was used to estimate the energy of the samples. Total carbohydrates were calculated using the following equation:

$$Carbohydrates(\frac{g}{100\;g}DW)=total\;solids-(protein+lipids+fibre+ash)$$

The chemical elemental analysis was determined by inductively coupled plasma mass spectrometry (ICP-MS; Agilent Technologies, Bellevue, WA, USA) after digestion with concentrated nitric acid (68% HNO_3_), and filtered and diluted 20 times with double distilled water (WP750, PG Instruments, Lutterworth, UK) to a total volume of 15 mL. For ICP-MS determinations, external standard calibration curves were performed by serially diluting multi-element standard stock solutions. This protocol was adapted from Mędyk et al., 2016 [58].

Additionally, we compared the nutritional and mineral composition of *T. arenaria* with that of *A. bisporus*, *L. edodes*, *P. ostreatus* (edible mushrooms) and *T. melanosporum* (edible truffle), and beef, pork and chicken meat. Data used for the other edible mushrooms and truffles were selected from studies published in international peer-review journals reporting the use of methodologies similar to those we used in our study. Therefore, we used one article for *T. arenaria*, three for *A. bisporus*, five for *L. edodes*, six for *P. ostreatus* and two for *T. melanosporum*. For beef, pork and chicken meat, one database and one article were consulted for each.

Finally, data on dietary reference intakes of nutrients and elements were compiled from Dietary Reference Intakes Datasets from the USA, Canada [59], and the EU [60,61,62,63]. The contribution of 100 g of dried and fresh *T. arenaria* to the daily intake of each nutrient and element was calculated considering the dietary reference intakes values previously compiled. Finally, we determined the Nutri-Score for *T. arenaria* based on its nutritional composition per 100 g of dry truffles. We used the nutritional content determined in this study, and complemented it with data on sugars and fatty acids from Tejedor-Calvo et al., 2021 [23]. To determine the Nutri-Score we used the recent algorithm made available by Sante Publique France (https://www.santepubliquefrance.fr/en/nutri-score (accessed on 9 August 2023)) [64].

### 2.3. Volatiles Profile by GC–MS

To showcase its unique bouquet of volatile organic compounds (VOCs), we collected (as previously described in Section 2.1.) and analysed *T. arenaria*’s samples for their VOCs profile. Furthermore, *T. arenaria*’s data was compared with that reported in the literature for the same other edible mushroom and truffle species previously described (*A. bisporus*, *L. edodes*, *P. ostreatus* and *T. melanosporum*).

Analysis of *T. arenaria*’s VOCs profile was performed using Headspace-Solid Phase Microextraction Gas Chromatography–Mass Spectrometry coupled to GC–MS (HS–SPME/GC–MS), adapted from the protocol reported by Splivallo and Ebeler (2015) [65]. The three fresh specimens were ground with a clean knife to small cubes of approximately 125,000 mm^3^, and accurately weighed in 1.5 mL tightly sealed glass vials. A pre-extraction was performed in the vial at 60 °C for 10 min, then the SPME fibre (PDMS/DVB65um) was implanted manually, and the volatile compounds were extracted at 60 °C for 30 min. Afterwards, the SPME fibre was removed and placed manually in the injection port of the GC–MS. The analysis of volatile compounds was conducted on an GC–MS-QP2010 (Shimadzu, Japan), with acquisition mode SCAN (35–600 *m/z*) and equipped with a TRB-5 MS column (Teknokroma, Barcelona, Spain). The injector and MS interface temperatures were both held at 250 °C. The analytic conditions were the following: the constant flow of helium in the column was kept at 1.0 mL min^−1^; the oven temperature was held at 40 °C for 10 min, then raised at a rate of 10 °C min^−1^ to 160 °C, and finally reached 260 °C with a rate of 50 °C min^−1^ and kept for 2 min. Blank GC–MS runs were performed during the analyses.

Finally, we compared the VOCs profile of *T. arenaria* with that of *A. bisporus*, *L. edodes, P. ostreatus* (edible mushrooms) and *T. melanosporum* (edible truffle). Data used for these edible mushrooms and truffle were selected from studies published in international peer-review journals reporting the use of methodologies similar to those we used in our study. Therefore, we used one article per each species.

### 2.4. First Steps in Developing a Non-Destructive and Rapid Identification Method for T. arenaria

**Figure 1:**
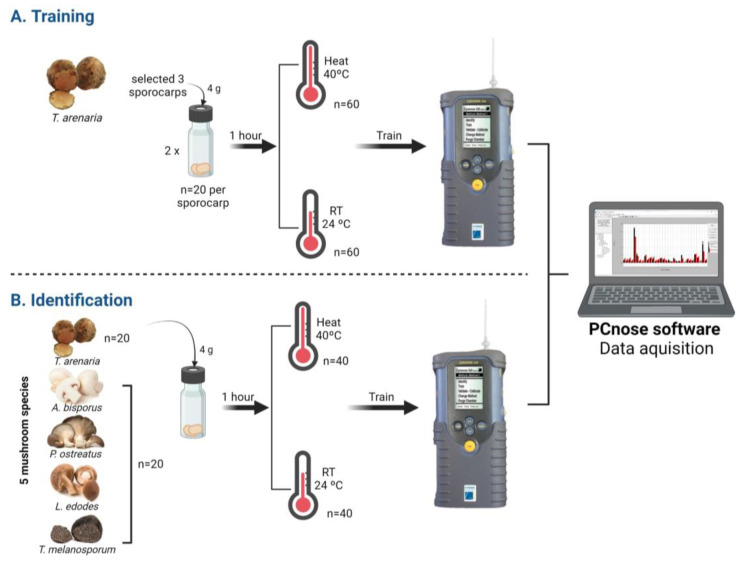
Schematic representation of the two phases of the non-destructive and rapid identification method for *T. arenaria* identification using the E-nose Cyranose-320. First, the e-nose was trained with *T. arenaria* samples (**A**), and then mushroom and truffle samples belonging to five species were tested for an accurate identification of *T. arenaria* (**B**). Two pre-analysis incubation temperatures were tested in both phases: samples were kept at room temperature (RT) or heated at 40 °C.

To test if *T. arenaria*’s unique VOCs profile could be applied in developing a non-destructive and rapid identification method for the early detection of *T. arenaria*, we used the eletronic nose Cyranose-320 (Sensigent, Pasadena, CA, USA). The Cyranose-320 is a portable e-nose equipped with a nanocomposite sensor array (32 nanosensors), an internal air sampling pump, and advanced pattern recognition algorithms. These technologies enable rapid detection and identification of substances based on their chemical profile as visualized by the smellprint [66]. Therefore, we specifically tested if the e-nose was capable of distinguishing *T. arenaria* from other edible mushroom and truffle species, and therefore guarantee this desert truffle’s authenticity. This was conducted in the following two phases (Figure 1):

#### 2.4.1. Phase 1: E-Nose Training

For the training process (Figure 1), *T. arenaria*’s samples were subjected to one of two pre-analysis incubation temperatures: (a) 40 °C treatment with samples incubated for 1 h at 40 °C; and (b) RT treatment with samples incubated for 1 h at room temperature (i.e., 24 °C). The samples used for analysing *T. arenaria*’s VOCs profile with the e-nose were clean as previously described, and were kept at 4 °C until analysis in the first 48 h post-harvest. Three fresh *T. arenaria* truffles were analysed separately. Two replicates with 4 g of *T. arenaria* were weighed and introduced in a 10 mL vial for each sporocarp and training method. The *T. arenaria* truffles were identified as: Terf1, Terf2 and Terf3. The Cyranose-320 was mounted on a tripod, which could be adjusted for inserting the e-nose needle into the vials for headspace reading. Ten readings per samples were performed. The e-nose was coupled to the computer and PCnose software was used to set the list of parameter settings of the Cyranose-320 (see Table S1), data acquisition, and analysis. To finish the training phase, an internal data cross-validation was used to assess the accuracy of sample classification in relation to their respective class labels, serving as a measure of effectiveness for the e-nose system [67].

#### 2.4.2. Phase 2: E-Nose Identification Accuracy

Similarly to what was conducted in the training phase, four grams of three fresh mushrooms were weighed and introduced in a 10 mL vial, and the two pre-analysis incubation temperatures were applied. Afterwards, each sample headspace was read with Cyranose-320 in the identification mode activated for the respective method trained (40 °C or RT pre-analysis incubation temperatures). Results were displayed in Cyranose-320 and recorded on the PCnose software. The results displayed in the Cyranose-320 are rated with asterisks, between one and five asterisks, accordingly to the identification quality performed. Regarding quality, only samples between three and five asterisks are considered acceptable results (i.e., acceptable, good and excellent, respectively). When the e-nose does not recognize the tested sample, “Confused” or “Unknown” will be displayed (Table S5).

### 2.5. Statistical Analysis

We used a principal component analysis (PCA) to analyse nutritional and mineral composition (based on fresh weight values) for *T. arenaria* determined in this study, and *A. bisporus*, *L. edodes* and *P. ostreatus* and fresh beef, pork and chicken meat with values from the literature. *T. melanosporum* was not included in the PCA because moisture content was not available on the selected literature and thus we were unable to express its nutritional and mineral composition for fresh samples. The PCA explored how *T. arenaria*’s nutritional and mineral composition compares to reference values for edible mushrooms and meat.

Pie charts and Venn diagram were performed to compare the VOCs profiles of *T. arenaria* with literature values for *A. bisporus*, *L. edodes*, *P. ostreatus* and *T. melanosporum* (http://bioinformatics.psb.ugent.be/webtools/Venn/ (accessed on 2 August 2023)).

Standardized data from the 32 sensors were analysed blinded to reference standard results using principal component analysis (PCA) to explore the sensors’ response to the two pre-analysis incubation temperatures (40 °C and RT). Differences between 40 °C and RT pre-analysis incubation temperatures were compared using the Kruskal–Wallis one-way analysis of variance. Multiple pairwise comparisons were performed using Dunn’s test (*p* < 0.05). All statistical analysis were performed using Microsoft Excel 2019/XLSTAT-Premium (Version 2021.4.1, Addinsoft, Inc., Brooklyn, NY, USA).

## 3. Results and Discussion

### 3.1. Showcasing T. arenaria’s Nutritional Value

The diverse array of nutrients found in mushrooms and truffles, including carbohydrates, proteins, lipids, minerals, fibre, and water, contribute to their potential positive effect on the human diet. The average moisture of the *Terfezia arenaria* samples was 77%, which is within the range reported for other desert truffles (Table 1) [20,21,68]. However, *T. arenaria*’s lipid concentration was slightly (2 to 8%) lower than that reported for other desert truffles [20,23,24,68], but similar to that reported for the commercial edible mushroom and truffle species *A. bisporus*, *L. edodes*, *P. ostreatus* and *T. melanosporum* [23,69,70,71,72]. Carbohydrates are the major nutrient category in edible mushrooms and truffles [73]. However, the concentrations determined in *T. arenaria* were lower than those reported for other desert truffles [20,24,68]. *T. arenaria*’s energy potential (387 kcal per 100 g of dry weight) was similar to that reported for the other edible mushrooms and truffles (Table 1) [21,23,69,70].

Furthermore, 18 mineral elements were identified in *T. arenaria* samples (Table 2), with potassium, phosphorus, sulphur, magnesium and calcium being the most abundant. Eight trace elements (iron > zinc > copper > manganese > chromium > molybdenum > selenium > nickel) and two nonessential elements (aluminum and lithium) were also identified. These mineral elements are critical for human health, and their intake must be carefully balanced to avoid health problems. Lithium (Li, 37 µg 100 g^−1^ dw) and selenium (Se, 50 µg 100 g^−1^ dw) are of particular importance to human health, due to their proprieties, i.e., antiviral, immunomodulatory, neuroprotective effects, and can be used to treat several mental health conditions [63,74]. Considering the dietary reference intakes (see Table S2), a balanced consumption of *T. arenaria* (especially dry) could contribute to a proper intake of these elements. In the case of lithium, this element is higher in *T. arenaria* than the other edible mushrooms and truffles, which could be an interesting characteristic in the development of new plant-based meat products based on this desert truffle.

Two trace elements with a detrimental health effect were identified in the *T. arenaria*, Arsenic (As, 10 µg 100 g^−1^ dw) and Barium (Ba, 32 µg 100 g^−1^ dw). The As value is similar to the values reported for *A. bisporus*, *L. edodes* and *Pleurotus ostreatus,* while the Ba was lower than that reported for *A. bisporus* and *L. edodes* [75,76] (Table 2), and other edible mushroom species [77]. Furthermore, according to Siwulski et al. (2021), the estimated daily intakes of these mushrooms, particularly *L. edodes*, are low and do not pose a health risk [76]. Also, considering the established dietary reference intakes for Arsenic (15 μg kg^−1^ body weight per day) and Barium (0.2 mg kg^−1^ body weight per day), the contribution of *T. arenaria* is residual when considering a consumption of 100 g of dry or fresh truffles (Table S2).

**Figure 2:**
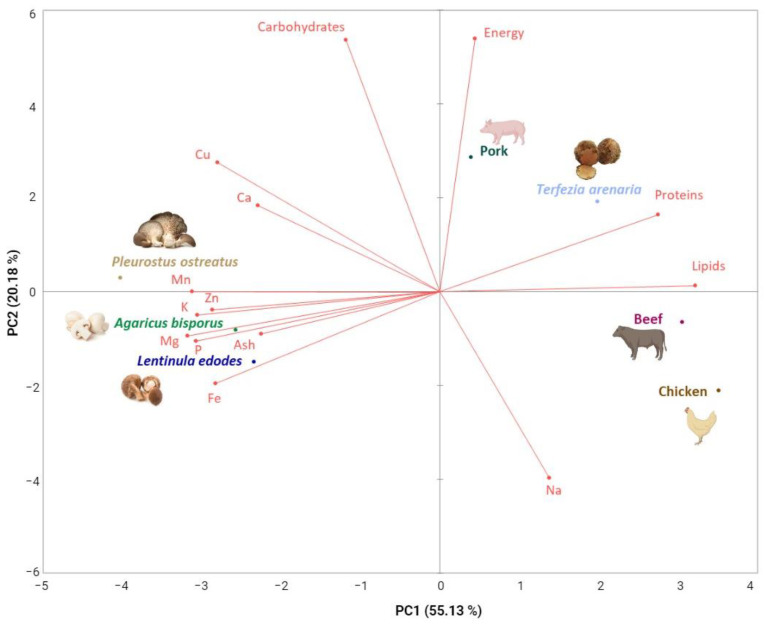
Fresh *T. arenaria*’s nutritional and mineral composition in relation to reference values reported for other fresh edible mushrooms and meat. Plot of the first two principal components of the PCA model built with common nutritional and mineral composition values for fresh *T. arenaria* determined in this study, and fresh *A. bisporus*, *L. edodes*, *P. ostreatus* and beef, pork and chicken meat from the literature.

The nutritional and mineral value of mushrooms is influenced by parameters such as the stage of development, the substrate where they grow, the geographic origins, and their genetic variability intra and interspecies [78]. *Terfezia arenaria* samples were harvested from different locations with different dominant forest species and at different times during a three-week harvest season. Although *T. arenaria* has a wider distribution area and fruits for a longer period, both its distribution area and fruiting period have been severely reduced by lower precipitation in autumn and spring (crucial for desert truffle fructification [9]) due to climate change and wildfires that destroy productive areas. However, when considering the nutritional values previously reported for other desert truffles, we consider that it is likely that the *T. arenaria*‘s nutritional and chemical composition we report here could represent this species’ composition. Nonetheless, *T. arenaria*‘s nutritional and mineral composition was similar to the most commercialized and appreciated species of mushrooms and truffles in the world, such as *A. bisporus*, *L. edodes*, *P. ostreatus* and *T. melanosporum* [79,80] (Table 2 and Table 3). Despite their similarities, *T. arenaria* have a closer resemblance to meat than the other edible mushrooms (Figure 2).

The Nutri-Score is a promising new front-of-pack nutrition labelling system that has the potential to improve population diets [81]. It is easy to understand, well-accepted by consumers, and can be effective in encouraging healthier food choices [82]. The Nutri-score algorithm showed that 100 g of dry *T. arenaria* has a Nutri-Score of A (i.e., the A score is the best nutritional score when applying the Nutri-Score), which indicates that this product has a very good nutritional profile. Products with an A score are typically low in calories, saturated fat, and sugar, and high in fibre and protein.

Our data on *T. arenaria*’s nutritional value can help explain why this desert truffle had, and still has, such an important role in the nourishment of rural populations, often serving as a meat substitute [11,15]. Indeed, poor rural populations in North Africa and Arab countries have used mushrooms as meat substitutes for centuries [11,12,15]. Furthermore, *T. arenaria* shares equally appealing properties with other edible commercial species (Table 1 and Table 2) as *T. arenaria*’s protein can range between 14 and 23 g per 100 g^−1^ dw, its carbohydrates can range between 67 and 77 g/100 dw, and its lipids can range between 2.2 and 5.1 g/100 dw (Table 1 and Table 2) [23]. *T. arenaria*’s protein value is similar to the average values for pork and beef meats (pork 13.2 g; beef 19.9 g), while the carbohydrates are higher (pork 2.4 g; beef 2.0 g) and the lipids are lower (pork 37.0 g; beef 4.2 g) [83]. These characteristics, together with the mineral composition, show that *T. arenaria* is suitable to be employed in new food products as a potential plant-based meat (Figure 2).

Indeed, as awareness on the adverse effects of meat consumption grows [84,85], there is a notable shift towards incorporating plant-based ingredients, such as mushrooms and truffles, into meat-based dishes [86,87]. This increasing acceptance reflects a growing interest in blending plant-based alternatives with traditional meat-based foods [88]. By incorporating mushrooms and truffles as blenders in meat products, over 7.5 million L of water can be saved per 10,000 portions of this product (made with 70% beef and 30% mushrooms) [88]. *A. bisporus*, *L. edodes* and *P. ostreatus* are among the most produced mushrooms species worldwide, and are already often used in these products [79,89].

**Table 1:** Nutritional composition and energy values for *T. arenaria* and other desert truffles, commercial mushrooms and meat (pork, beef and chicken). Values for *T. arenaria* were determined in the present study while values for the other mushrooms, truffles and meat were determined in other studies.

		Moisture	Ash	Proteins	Lipids	Carbohydrates	Fibre	Energy	References
		% fw	g/100 g	g/100 g	g/100 g	g/100 g	g/100 g	kcal/100 g	
Desert Truffles	*Terfezia arenaria* ^a^	77	7.3	14	2.2	67	10	387	This study
		n.a	4.3	23	5.1	77	n.a	394	[23]
	*Terfezia claveryi* ^a^	73	4.3	16	7.0	65	8	n.a	[68]
		83	15.3	32	2.8	46	n.a	338	[21]
	*Terfezia boudieri* ^a^	n.a	12.9	17	6.4	60	4	n.a	[24]
		78	4.5	26	8.0	62	n.a	n.a	[20]
	*Terfezia olbiensis* ^a^	80	15.3	36	3.2	48	n.a	366	[21]
Commercial mushrooms	*Agaricus bisporus* ^a^	91	12.7	19	2.0	67	10	360	[69]
		90	9.4	25	2.3	64	n.a	374	[70]
	*Pleurotus ostreatus* ^a^	91	7.8	18	2.6	71	14	382	[69]
		n.a	9.3	9	1.3	70	11	n.a	[71]
		89	6.7	13	2.5	78	n.a	383	[70]
	*Lentinula edodes* ^a^	n.a	3.8	18	0.9	30	32	264	[72]
		94	6.7	16	1.8	74	15	382	[69]
		88	7.4	17	2.1	73	n.a	381	[70]
	*Tuber melanosporum* ^a^	n.a	0.0	22	2.3	75	n.a	411	[23]
Meat	*Pork* ^b^			13	37.0	2.4	n.a	390	[83]
		64	0.9	18	17.5	n.a	n.a	228	[90]
	*Beef* ^b^			20	4.2	2.0	n.a	126	[83]
		63	0.8	18	19.4	n.a	n.a	243	[90]
	*Chicken* ^b^			19	1.3	9.4	n.a	167	[83]
		75	1.0	18	7.2	n.a	n.a	133	[90]

^a^ mushroom values presented for dry weight, except for moisture (% fresh weight). ^b^ meat values presented for fresh weight; n.a—data not available.

**Table 2:** Mineral content [mg kg^−1^ dw] for *T. arenaria* and other desert truffles, commercial mushrooms and meat (pork, beef and chicken). Values for *T. arenaria* were determined in the present study while values for the other mushrooms, truffles and meat were determined in other studies.

Minerals	*Terfezia* *arenaria*	*Agaricus* *bisporus*	*Lentinula edodes*	*Pleurotus* *ostreatus*	*Tuber* *melanosporum*	Pork	Beef	Chicken
Major essential elements								
Ca	26	580	438	730	817	60	70	60
K	3695	38,400	21,700	14,244	7356	3180	2730	3020
Mg	128	1300	1330	2800	241	190	164	205
Na	23	491	144	35	67	540	550	630
P	1407	8210	4080	6204	2678	1730	1440	1660
S	299	n.a	n.a	n.a	n.a	n.a	n.a	n.a
Essential trace elements								
Cr	0.9	7.0	0.3	n.a	n.a	n.a	n.a	n.a
Cu	6.6	34.2	7.1	39	18	0.7	0.6	0.4
Fe	19.6	49.4	35.7	130	12	7.9	19.6	5.9
Mn	1.4	6.6	19.3	14	1	<0.125	<0.125	0.1
Mo	0.6	0.3	0.2	<0.01	n.a	n.a	n.a	n.a
Ni	0.1	0.7	0.1	0.7	n.a	n.a	n.a	n.a
Se	0.5	1.7	1.1	0.3	n.a	n.a	n.a	n.a
Zn	11.0	51.5	76.3	110.4	37	22.3	38.5	11.8
Non-essential elements								
Al	10.7	17.9	5.8	n.a	n.a	n.a	n.a	n.a
Li	0.4	<0.1	0.1	0.3	n.a	n.a	n.a	n.a
Elements with detrimental health effects								
As	0.1	0.3	0.5	<0.1	n.a	n.a	n.a	n.a
Ba	0.3	2.8	1.7	n.a	n.a	n.a	n.a	n.a
References	This study	[75]	[76]	[91,92,93]	[94]	[90]	[90]	[90]

n.a—data not available.

**Table 3:** List of the volatile organic compounds (VOCs) detected in *T. arenaria* using GC–MS. The VOCs are listed according to their abundance (i.e., area). The values represent the mean relative peak areas (expressed as % from total peak areas) and retention times (RT). Information on each VOC’s classification, metabolic pathway and odor is also presented (n = 3).

Formula	RT	Area (%)	Name of Compounds	Funtional Groups	Pathway	Odor
C_8_H_16_O	15.847	57.21	1-Octen-3-ol	Alcohols	Lipoxygenase–linoleic acid	Mushroom like
C_8_H_16_O	16.007	12.86	3-Octanone	Ketones	Lipoxygenase–linoleic acid	Green apple-like
C_6_H_12_O	7.412	4.29	Hexanal	Aldehydes	Lipoxygenase–linoleic acid	Green, grassy
C_8_ H_16_ O	17.952	3.22	(Z)-2-Octen-1-ol	Alcohols	Lipoxygenase–linoleic acid	Green, citrus
C_10_H_16_	14.379	1.87	α-Pinene	Terpenes	Monoterpenoid biosynthesis	Woody, resinous
C_8_H_18_O	16.256	1.54	3-Octanol	Alcohols	Lipoxygenase–linoleic acid	Floral, fatty
C_8_H_14_O	15.658	1.38	(5Z)-Octa-1,5-dien-3-ol	Alcohols	Lipid metabolism	Sweet or floral
C_8_ H_16_ O_2_	16.549	0.98	Pentyl propanoate	Ester	n.a	Fruity, sweet
C_8_ H_14_ O	17.735	0.86	I-2-Octenal	Aldehydes	Lipoxygenase–linoleic acid	Fatty, nutty
C_21_H_41_IO_2_	24.933	0.39	Propionic acid, 3-iodo-, octadecyl ester	Ester	n.a	n.a
C_10_ H_16_	17.086	0.35	Limonene	Terpenes	Monoterpenoid biosynthesis	Citrus
C_8_H_9_N	18.411	0.32	Pyridine, 5-ethenyl-2-methyl-	Other compounds	n.a	Pungent, fish-like
C_18_H_37_ClO_2_S	25.156	0.3	1-Octadecanesulphonyl chloride	Other compounds	n.a	Strong and pungent
C_21_H_42_O_2_	25.127	0.29	Henicosanoic acid	Other compounds	n.a	Odorless
C_32_H_66_	25.155	0.28	Dotriacontane	Hydrocarbons	n.a	Odourless
C_8_ H_16_	17.479	0.26	Caprylene (1-octene)	Hydrocarbons	n.a	Petroleum-like
C_8_H_15_NO_3_	20.904	0.23	2-Octanone, 1-nitro-	Ketones	n.a	Sweet
C_12_H_24_O_3_	23.046	0.22	Propanoic acid, 2-methyl-, 3-hydroxy-2,2,4-trimethylpentyl ester	Ester	n.a	Mild, fruity or sweet
C_8_H_8_O	17.417	0.21	Benzeneacetaldehyde	Aldehydes	Phenylalanine metabolism	Sweet, floral
C_14_ H_30_	24.692	0.21	Tetradecane	Hydrocarbons	n.a	Gasoline-like to odorless
C_14_ H_30_	24.692	0.21	Eicosane-7-hexyl	Hydrocarbons	n.a	n.a
C_13_ H_22_ O_3_ Si_2_	18.831	0.19	Benzaldehyde, 2,5-bis[(trimethylsilyl)oxy]	Aldehydes	n.a	n.a
C_16_H_34_	24.134	0.15	Hexadecane	Hydrocarbons	Fatty acid degradation	Odourless
C_6_H_13_ClO	23.201	0.14	Chlorohexanol	Alcohols	n.a	Odorless
C_10_H_22_	24.654	0.13	3,3,5-Trimethylheptane	Hydrocarbons	n.a	Gasoline-like
C_7_H_7_NO_2_	24.979	0.13	Anthranilic acid	Other compounds	L-tryptophan-kynurenine	Odorless
C_9_H_18_O	20.273	0.12	Nonanal	Aldehydes	n.a	Fruity, waxy
C_8_H_10_O_2_	23.461	0.11	Tyrosol	Other compounds	Tyrosine metabolism	Floral, phenolic
C_16_H_32_	23.812	0.1	1-Dodecanol	Alcohols	n.a	Waxy, fatty
C_20_H_41_Cl	25.336	0.1	1-chloroeicosane	Hydrocarbons	n.a	n.a
C_13_H_22_O	23.68	0.09	Geranylacetone	Ketones	Ketone Body Metabolism	Sweet, floral, fruity
C_20_ H_42_	23.201	0.08	Eicosane	Hydrocarbons	n.a	Odourless

n.a—data not available.

### 3.2. Volatiles Profile by GC–MS

Volatile profiles, particularly in mushrooms and truffles, are crucial in determining their characteristic odours and strongly influence consumers’ preferences. *T. arenaria*’s distinct volatile profile serves as a key characteristic and significantly impacts consumers’ preferences.

Thirty-two VOCs were identified in *T. arenaria* fresh samples, i.e., eight hydrocarbons, six alcohols, five aldehydes, three ketones, three esters, two terpenes, and five other compounds. Among them, the most abundant were the eight carbon (C-8) compounds and Hexanal, with 1-octen-3-ol being the main volatile (64%) in *T. arenaria* (see Tables S3 and S4). 1-Octen-3-ol is generally referred to as the mushroom alcohol and is one of the most abundant VOCs produced by fungi [95,96]. This is consistent with the fact that C-8 compounds are the main volatile components found in several edible mushrooms and truffles [97,98,99,100] (Tables S3 and S4). Despite using similar methodologies, Harki et al. (2010) identified 27 VOCs in *T. arenaria*, but only three compounds were common with our study (nonanal, 3-octanone and 2-octenal) [34]. Both internal factors (e.g., maturity stage, tissue specificity and postharvest storage [101]) and external factors (e.g., place of origin, interaction with microorganisms [102]), result in distinct metabolic processes within the fungi, which alters their VOCs profile. The main volatiles identified in *T. arenaria* were C-8 compounds resulting from the breakdown of fatty acids (such as linoleic acid) by lipoxygenase and related enzymes (Table 3), which is in agreement with the evidence that the umami taste, so characteristic of mushrooms, is associated with the fatty acid metabolism [103]. Lipoxygenases are pivotal in the biosynthesis of leukotrienes, associated with various inflammatory conditions such as cancer, arthritis, asthma, and allergies [104]. Given their role in these disease processes, lipoxygenase inhibitors are being explored as potential therapeutic options for preventing and managing these inflammatory disorders [105,106]. Notably, certain mushroom extracts have demonstrated the ability to inhibit these enzymes, offering potential health benefits [105,107,108]. These extracts have been used to enhance the nutritional value of pasta, contributing to healthier products [107,108]. Exploring the potential inhibitor of *Terfezia* may be necessary for incorporating this product in food formulations.

**Figure 3:**
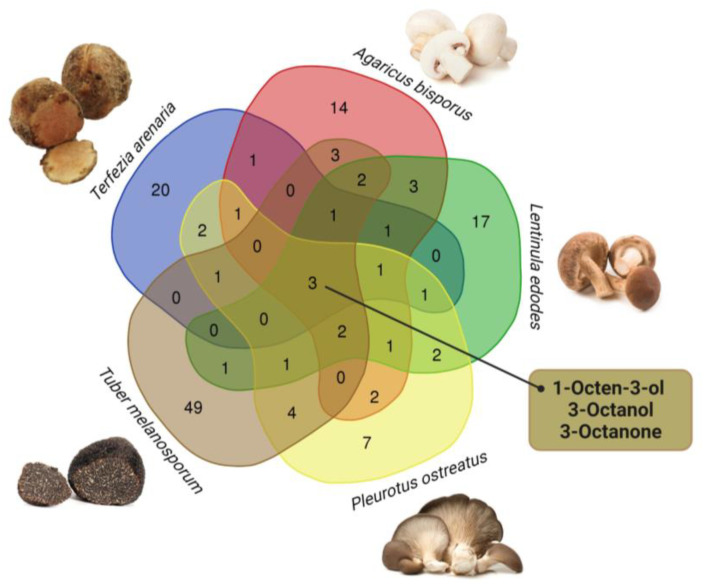
A Venn diagram comparing *T. arenaria*’s VOCs profile with that reported in the literature for *A. bisporus*, *L. edodes*, *P. ostreatus* and *T. melanosporum* (http://bioinformatics.psb.ugent.be/webtools/Venn/ (accessed on 2 August 2023)).

Nine VOCs identified in *T. arenaria* (i.e., 1-octen-3-ol, 3-octanol, 3-octanone, 2-octenal, hexanal, nonanal, benzeneacetaldehyde, eicosane, limonene and α-pinene) had been previously reported for the desert truffles *T. boudieri* and *T. claveryi* [35,36,109]. These compounds are also prevalent in commercial edible mushrooms and truffles such as *A. bisporus*, *L. edodes*, *P. ostreatus* and *T. melanosporum* [98,99,100,110]. On the other hand, 18 VOCs detected in *T. arenaria,* had not been reported for other *Terfezia* spp. or *A. bisporus*, *L. edodes*, *P. ostreatus* and *T. melanosporum* (Table S4). Additionally, a review of the reported VOCs composition of these species revealed that only three compounds are common between *T. arenaria* and these four commercial species (Figure 3). These VOCs, which are C-8 compounds (i.e., 1-octen-3-ol, 3-octanol, 3-octanone), are abundant in *A. bisporus*, *L. edodes*, *P. ostreatus* and *T. arenaria* and greatly contribute to their floral and green aromas [37] (Table 2 and Table S3). They are also present in *T. melanosporum*, but in lower quantities [18,102].

**Figure 4:**
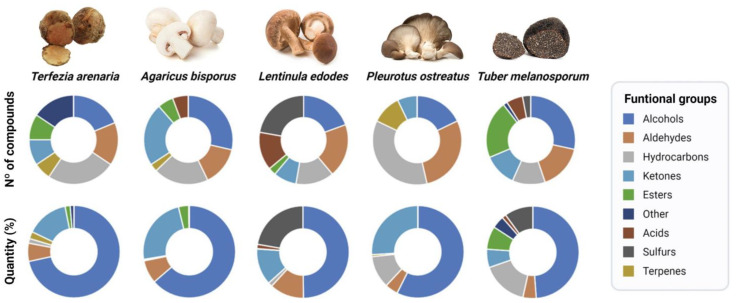
Aromatic profiles of the five edible mushroom and truffles species as shown by the number and identity of the functional groups of compounds and their relative proportion. The pie charts present the number of identified compounds and their relative proportion (%) per group in each mushroom and truffle species.

When comparing *T. arenaria*’s aroma profile with that from other edible mushrooms and truffles (*A. bisporus*, *L. edodes*, *P. ostreatus*, and *T. melanosporum*) (Figure 3, Table S4), *T. arenaria* exhibits a distinct composition. Figure 4 highlights potential differences between *T. arenaria* and the other edible mushrooms and truffles in terms of the number and quantity of compounds per main functional group. In *T. arenaria*, there is a higher presence of alcohol compounds compared to *A. bisporus* and *T. melanosporum*, although it is less diverse in terms of the number of compounds. Unlike *L. edodes* and *T. melanosporum*, *T. arenaria* does not contain detectable acids or sulphur compounds, making it stand apart in its aromatic profile. The latter two species have a greater variety of compounds, indicating a more complex volatile profile than *T. arenaria*.

On the other hand, *A. bisporus* and *P. ostreatus* demonstrate simpler volatile profiles, with only five main functional groups identified. Among all the analysed edible mushrooms and truffles, *A. bisporus* appears to have the most similar aromatic profile to *T.* arenaria. Despite the high similarity between the VOCs profiles of *T. arenaria* and *A. bisporus* (Figure 4), the e-nose successfully distinguished between the volatile profiles these fungal species [41,52,53,54,55].

### 3.3. First Steps in Developing a Non-Destructive and Rapid Identification Method for T. arenaria

Electronic noses have been coupled with gas chromatography–mass spectrometry (GC–MS) to analyse aromas in various food products [111]. This technique has gained prominence due to its versatility and speed of response. Although GC–MS is a more precise and accurate technique, it is also more complex, expensive, and time-consuming. For example, in this work, analysing only three samples using GC–MS required one week, including sample preparation, sample reading, and data processing. In contrast, using an e-nose, after the initial equipment training (which takes about 4 h), a quick response can be obtained within one to two hours (including sample preparation, incubation, and equipment reading).

#### 3.3.1. Phase 1: Electronic Nose Training

**Figure 5:**
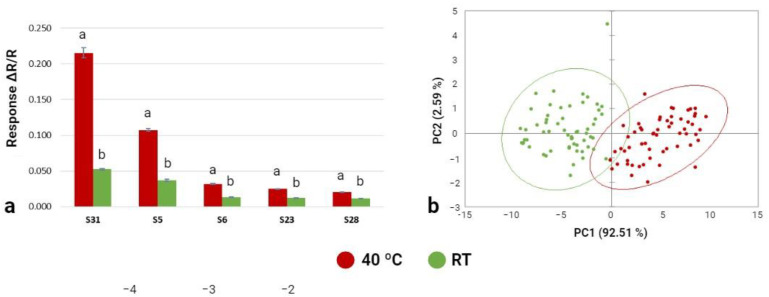
Effect of the pre-analysis incubation temperature (40 °C and RT) on the Cyranose-320 sensor’s response to *T. arenaria* fresh samples. (**a**) Histogram of the 5 sensors showing larger responses to *T. arenaria* samples. Different letters show a significant effect (*p* < 0.05) of the pre-analysis incubation temperature on the sensors responses. (**b**) Plot of the first two principal components of the PCA model built with the Cyranose-320 data related to *T. arenaria* samples at 40 °C and RT.

As the Cyranose-320 e-nose showed sensitivity to the pre-analysis incubation temperature (40 °C or 24 °C), temperature influenced the volatile compounds emitted by *T. arenaria* fresh samples (Figure 5). The Cyranose-320 correctly classified 73% of the *T. areanaria* samples incubated at room temperature, and 81% of the *T. areanaria* samples incubated at 40 °C. All 32 sensors of the Cyranose-320 e-nose signalled the emitted VOCs for pre-analysis incubation temperatures (40 °C and 24 °C). From those, five sensors had major responses in the two pre-analysis incubation temperatures (i.e., S5, S6, S23, S28, and S31), and sensor number 31 showed the highest sensitivity (Figure 5a). The pre-analysis incubation temperature influenced these sensors (i.e., S5, S6, S23, S28, and S31; *p* < 0.001), with higher responses at the higher temperature. The first two principal components (PC1 and PC2) explained 95.1% of the total variance (92.5% and 2.6%, respectively), which means that the incubation temperature has a significant effect on the volatile compounds emitted by *T. arenaria* (Figure 5b). Similar results were reported for *A. bisporus* (92% and 99%) stored at different temperatures [51]; *Tuber indicum* (100%) under different drying processes [50]; and *Thricholoma matsutake* (90%) from different provenience regions. This confirms the importance of the pre-analysis temperature (i.e., storage temperature) and the sensitivity of the e-nose methodology to detect the volatile profile of different mushroom and truffle species. The score plot (Figure 5b) showed that the aroma profile of *T. arenaria* subjected to different pre-analysis incubation temperatures can be discriminated and the two clusters were very similar in terms of their volatile compound content (Figure 5a).

#### 3.3.2. Phase 2: E-Nose Identification Accuracy

**Figure 6:**
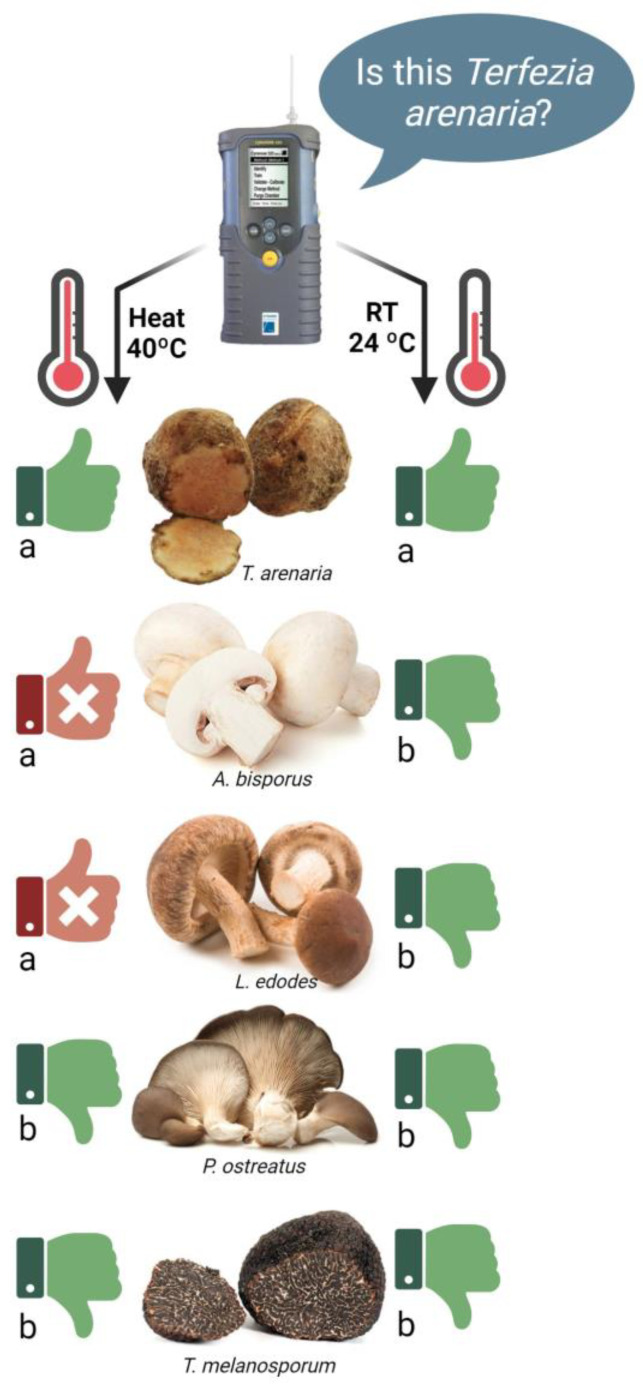
Effect of the pre-analysis incubation temperature (40 °C and RT) on the e-nose Cyranose-320 capacity to accurately identify *T. arenaria*, and distinguish it from *A. bisporus*, *L. edodes*, *P. ostreatus* and *T. melanosporum*. The “thumbs up” symbol represents the cases when the e-nose identified the sample as being *T. arenaria*, while the “thumbs down” symbol represents the cases when the e-nose identified the sample as not being *T. arenaria*. Green thumbs indicate an accurate identification, while red thumbs indicate an inaccurate identification by the e-nose. Different letters show a significant effect (*p* < 0.05) of the pre-analysis incubation temperature on the identification accuracy for each species.

Based on their volatile profile, the e-nose Cyranose-320 accurately recognized the *T. arenaria* samples (Terf1, Terf2 or Terf3) and rated their result with stars (Table S5). Although all *T. arenaria* samples were identified as one of the trained volatile profiles in both pre-analysis incubation temperatures, the pre-analysis incubation temperature influenced the identification accuracy. So, when the pre-analysis incubation was performed at room temperature, the identification of 80% of the samples was acceptable or excellent, while when the pre-analysis incubation was performed at 40 °C, only 45% of the samples were acceptable or excellent, and considered accurately identified (Table S5). Despite this trend, the pre-analysis incubation temperature did not affect the *T. arenaria* samples’ identification accuracy (*p* < 0.05) (Figure 6). Nevertheless, pre-analysis incubation at room temperature improved the e-nose’s capacity to distinguish *T. arenaria* samples from those of the other edible mushrooms and truffles (*A. bisporus*, *L. edodes*, *P. ostreatus* and *T. melanosporum*) (Figure 6).

Indeed, when the pre-analysis incubation was performed at 40 °C, some samples were misclassified as *T. arenaria* (Figure 6), specifically some *A. bisporus* and *L. edodes* samples were identified with a 100% probability of being *T. arenaria* (5 stars). The pre-analysis incubation temperature affected the e-nose’s capacity to distinguish between *T. arenaria* and *A. bisporus* and *L. edodes* but not *P. ostreatus* and *T. melanosporum* (*p* < 0.05).

Most of the studies reporting the use of the e-nose technology on mushrooms and truffles focused on sample quality as influenced by dehydration [48,49,50], shelf life and packaging [43,51], and maturation process [45]. Some studies also explored the differentiation of species by analysing their volatile profiles using the e-nose [41,52,53,54,55]. By applying the e-nose methodology for a rapid and non-destructive accurate identification of *T. arenaria* samples, we demonstrate our approach’s potential for mushrooms and truffles identification during harvest. It is important to keep in mind that *T. arenaria* is traditionally harvested near the host plant (*Tuberaria guttata)*, with the collector using a pointed stick to repeatedly explore the soil until a truffle is detected and extracted [13]. Therefore, the development of a tool based on our non-destructive and rapid methodology could contribute to the early detection of *Terfezia* truffles in the field, potentially discriminating between maturity stages and sample quality, which would contribute to the much-needed technology to boost *Terfezia*’s sustainable production and ensure its reliable identification. Furthermore, this could be useful for other truffles whose belowground development makes it difficult to detect them and distinguish maturity stages and quality [15]. As the VOCs emitted by mushrooms and truffles are important to the food industry, especially for developing new food products or even for new tools that could contribute to food security, our study contributes to unlock many possibilities for using this delicacy (*T. arenaria*) in the food industry worldwide.

## 4. Conclusions

From a nutritional standpoint, *T. arenaria* is a well-balanced food, rich in carbohydrates, fibres, and proteins, while containing a low-fat content. It is also a good source of minerals, including lithium, selenium, and iron. Furthermore, it has a unique aroma dominated by the C8-compounds produced in the lipoxygenase pathway. Twenty nine new volatile organic compounds (VOCs) were identified for *T. arenaria*, from which the C8-compounds produced in the lipoxygenase pathway were predominant. Further analysis is required to understand the variations attributed to the specific internal and external factors and how these regulate fatty acid metabolism. In addition to the importance of defining *T. arenaria*’s aromatic profile, this metabolic pathway is also related to the umami taste, essential for the development of plant-based meat.

The e-nose Cyranose-320 accurately identified *T. arenaria* samples (especially when samples were incubated at room temperature before analysis) and was able to distinguish *T. arenaria* from other edible mushrooms and truffles (*A. bisporus*, *L. edodes*, *P. ostreatus* and *T. melanosporum*). The E-nose Cyranose-320 was more accurate when mushroom and truffle samples were pre-incubated at room temperature than at 40 °C. Our data point the e-nose’s great potential as a rapid and non-destructive detection tool for identifying *T. arenaria* and possibly other mushroom and truffle species. However, larger datasets (more samples and in different maturity stages) are necessary to fine-tune the parameter settings of the Cyranose-320 and optimize the identification process.

Altogether, *T. arenaria* is a nutritious and sustainable food that has the potential to be used in a variety of new food products. It is a good source of protein and minerals, and it has a Nutri-Score of A. *T. arenaria* could be used as a meat substitute or as an ingredient in plant-based meat products. Studying the nutritional composition and volatile profile of *T. arenaria* provides valuable insights into its potential as a nutritious food source. The application of the electronic nose technology enhances our ability to identify and authenticate the unique aroma profiles of these desert truffles. Moreover, this study unveils the e-nose’s potential for the early detection of *T. arenaria* in the field, which could contribute to the sustainable production of this delicacy. The electronic nose could also be used to distinguish between maturity stages and quality of *T. arenaria*, which would be valuable for ensuring the quality of this food product. This knowledge contributes to advancements in food science and technology, supporting the development of quality control measures and ensuring the authenticity of food products in the market.

## Data Availability

The datasets generated for this study are available on request to the corresponding author.

## Supplementary Materials

The following supporting information can be downloaded at: https://www.mdpi.com/article/10.3390/foods12193527/s1, Figure S1: Map of the sampling areas of *Tefezia arenaria* collected during this study, and forest-dominant species per area. Table S1: Parameters settings for using the Cyranose-320; Table S2: Dietary Reference Intakes for nutrients and elements; Table S3: Comparison of the abundance of the main VOCs identified in *T. arenaria* and in the other edible mushroom and truffle species; Table S4: Characterization of the VOCs identified in *T. arenaria*, and in the other edible mushroom and truffle species (*A. bisporus*, *L. edodes*, *P. ostreatus* and *T. melanosporum*); Table S5: Results and rates of Cyranose-320 identification of *T. arenaria*, *A. bisporus*, *L. edodes*, P. ostreatus and *T. melanosporum* with 40 °C and RT pre-analysis incubation temperatures.

## References

[1] Köberle A.C. (2022). Food Security in Climate Mitigation Scenarios. Nat. Food.

[2] Ferreira I., Corrêa A., Cruz C. (2023). Sustainable Production of Ectomycorrhizal Fungi in the Mediterranean Region to Support the European Green Deal. Plants People Planet.

[3] Boa E. (2004). Wild Edible Fungi. A Global Overview of Their Use and Importance to People. Non-Wood Forest Products.

[4] Ferreira I., Dias T., Cruz C. (2023). The Potential of Ectomycorrhizal Fungi to Modulate below and Aboveground Communities May Be Mediated by 1-Octen-3-Ol. J. Fungi.

[5] de Frutos P. (2020). Changes in World Patterns of Wild Edible Mushrooms Use Measured through International Trade Flows. For. Policy Econ..

[6] FAOSTAT. https://www.fao.org/faostat/en/#home.

[7] Pérez-Moreno J., Guerin-Laguette A., Rinaldi A.C., Yu F., Verbeken A., Hernández-Santiago F., Martínez-Reyes M. (2021). Edible Mycorrhizal Fungi of the World: What is Their Role in Forest Sustainability, Food Security, Biocultural Conservation and Climate Change?. Plants People Planet.

[8] Pérez-Moreno J., Mortimer P., Xu J., Karunarathna S., Li H., Pérez-Moreno J., Mortimer P., Xu J., Karunarathna S., Li H. (2021). Global Perspectives on the Ecological, Cultural and Socioeconomic Relevance of Wild Edible Fungi. Stud. Fungi.

[9] Andrino A., Navarro-Ródenas A., Marqués-Gálvez J.E., Morte A. (2019). The Crop of Desert Truffle Depends on Agroclimatic Parameters during Two Key Annual Periods. Agron. Sustain. Dev..

[10] Oliach D., Morte A., Sánchez S., Navarro-Ródenas A., Marco P., Gutiérrez A., Martín-Santafé M., Fischer C., Albisu L.M., García-Barreda S., Sánchez-González M., Calama R., Bonet J.A. (2020). Las Trufas y Las Turmas. Los Productos Forestales no Madereros en España: Del Monte a la Industria.

[11] Bradai L., Neffar S., Amrani K., Bissati S., Chenchouni H. (2015). Ethnomycological Survey of Traditional Usage and Indigenous Knowledge on Desert Truffles among the Native Sahara Desert People of Algeria. J. Ethnopharmacol..

[12] Shavit E. (2014). The History of Desert Truffle Use.

[13] Chevalier G., Kagan-Zur V., Roth-Bejerano N., Sitrit Y., Morte A. (2014). The European Desert Truffles. Desert Truffles. Soil Biology.

[14] Morte A., Arenas F., Marqués-Gálvez J.E., Andrino A., Guarnizo Á.L., Gutiérrez A., Berná L.M., Pérez-Gilabert M., Rodríguez A., Navarro-Ródenas A., Al-Khayri J.M., Jain S.M., Johnson D.V. (2021). Desert Truffles (*Terfezia* spp.) Breeding. Advances in Plant Breeding Strategies: Vegetable Crops.

[15] Shavit E. (2008). Truffles Roasting in the Evening Fires. Pages Hist. Desert Truffles Fungi.

[16] Gadallah M.G.E., Ashoush I.S. (2016). Value Addition on Nutritional and Sensory Properties of Biscuit Using Desert Truffle (*Terfezia claveryi*) Powder. Food Nutr. Sci..

[17] Najjaa H., Abdelkbir R., Ben Arfa A., Doria E., Tlili H., Zouari N., Neffati M. (2021). Improved Sensory Quality and Antioxidant Capacity of Wheat Bread Supplemented with the Desert Truffle *Terfezia boudieri* Flour. Anal. Lett..

[18] Khalifa S.A.M., Farag M.A., Yosri N., Sabir J.S.M., Saeed A., Al-Mousawi S.M., Taha W., Musharraf S.G., Patel S., El-Seedi H.R. (2019). Truffles: From Islamic Culture to Chemistry, Pharmacology, and Food Trends in Recent Times. Trends Food Sci. Technol..

[19] Martínez-Tomé M., Maggi L., Jiménez-Monreal A.M., Murcia M.A., Marí J.A.T. (2014). Nutritional and Antioxidant Properties of Terfezia and Picoa.

[20] Hamza A., Zouari N., Zouari S., Jdir H., Zaidi S., Gtari M., Neffati M. (2016). Nutraceutical Potential, Antioxidant and Antibacterial Activities of *Terfezia boudieri* Chatin, a Wild Edible Desert Truffle from Tunisia Arid Zone. Arab. J. Chem..

[21] Kıvrak İ. (2015). Analytical Methods Applied to Assess Chemical Composition, Nutritional Value and In Vitro Bioactivities of *Terfezia olbiensis* and *Terfezia claveryi* from Turkey. Food Anal. Methods.

[22] Al Obaydi M.F., Hamed W.M., Al Kury L.T., Talib W.H. (2020). *Terfezia boudieri*: A Desert Truffle With Anticancer and Immunomodulatory Activities. Front. Nutr..

[23] Tejedor-Calvo E., Amara K., Reis F.S., Barros L., Martins A., Calhelha R.C., Venturini M.E., Blanco D., Redondo D., Marco P. (2021). Chemical Composition and Evaluation of Antioxidant, Antimicrobial and Antiproliferative Activities of *Tuber* and *Terfezia* Truffles. Food Res. Int..

[24] Ahmed A.A., Mohamed M.A., Hami M.A. (1981). Libyan Truffles *Terfezia boudieri* Chatin: Chemical Composition and Toxicity. J. Food Sci..

[25] Amara K., Reis F.S., Barros L., Skhiri F., Martins A., Ferreira I.C.F.R. Nutritional Values, Chemical Characterization and Cytotoxicity in Human Tumor Cell Lines of Desert Truffles. Proceedings of the 8th Journées Scientifiques Internationales sur la Valorisation des Bioressources.

[26] Benaceur F., Chaibi R., Berrabah F., Neifar A., Leboukh M., Benaceur K., Nouioua W., Rezzoug A., Bouazzara H., Gouzi H. (2020). Purification and Characterization of Latent Polyphenol Oxidase from Truffles (*Terfezia arenaria*). Int. J. Biol. Macromol..

[27] Harir M., Bendif H., Yahiaoui M., Bellahcene M., Zohra F., Rodríguez-Couto S. (2019). Evaluation of Antimicrobial Activity of *Terfezia arenaria* Extracts Collected from Saharan Desert against Bacteria and Filamentous Fungi. 3 Biotech.

[28] Moreno G., Alvarado P., Manjón J.L. (2013). Hypogeous Desert Fungi. Desert Truffles: Phylogeny, Physiology, Distribution and Domestication.

[29] Ammarellou A., Wang Y., Nematzadeh G., Tajick M. (2014). Non-Mediterranean Asian Desert Countries.

[30] Dafri A., Beddiar A. (2018). Morphological Characterisation of the Mycorrhizal Symbiosis between *Tuberaria guttata* (L.) Fourr and *Terfezia arenaria* (Moris) Trappe. Symbiosis.

[31] Brenko A., Vidale E., Oliach D., Marois O., Andrighetto N., Stara K., de Aragón J.M., Bonet J.A. (2022). Short Communication: Edible Wild Mushrooms of the Northern Mediterranean Area–Sectorial Analysis and Future Perspectives. For. Syst..

[32] Egli S., Peter M., Buser C., Stahel W., Ayer F. (2006). Mushroom Picking Does Not Impair Future Harvests–Results of a Long-Term Study in Switzerland. Biol. Conserv..

[33] Peintner U., Schwarz S., Mešić A., Moreau P.-A., Moreno G., Saviuc P. (2013). Mycophilic or Mycophobic? Legislation and Guidelines on Wild Mushroom Commerce Reveal Different Consumption Behaviour in European Countries. PLoS ONE.

[34] Harki E., Farah A., Bouseta A. (2010). Volatile Compounds from Four Species of Moroccan Truffles. Vice Ed. Chief Vice Redacteur Chef.

[35] Farag M.A., Fathi D., Shamma S., Shawkat M.S.A., Shalabi S.M., El Seedi H.R., Afifi S.M. (2021). Comparative Metabolome Classification of Desert Truffles *Terfezia claveryi* and *Terfezia boudieri* via Its Aroma and Nutrients Profile. LWT.

[36] Kamle M., Bar E., Lewinsohn D., Shavit E., Roth-Bejerano N., Kagan-Zur V., Barak Z., Guy O., Zaady E., Lewinsohn E. (2017). Characterization of Morphology, Volatile Profiles, and Molecular Markers in Edible Desert Truffles from the Negev Desert. J. Agric. Food Chem..

[37] Zhu R., Wen Y., Wu W., Zhang L., Salman Farid M., Shan S., Wen J., Farag M.A., Zhang Y., Zhao C. (2022). The Flavors of Edible Mushrooms: A Comprehensive Review of Volatile Organic Compounds and Their Analytical Methods. Crc Cr Rev Food Sci..

[38] Mustafa A.M., Angeloni S., Nzekoue F.K., Abouelenein D., Sagratini G., Caprioli G., Torregiani E. (2020). An Overview on Truffle Aroma and Main Volatile Compounds. Molecules.

[39] Lubes G., Goodarzi M. (2018). GC–MS Based Metabolomics Used for the Identification of Cancer Volatile Organic Compounds as Biomarkers. J. Pharm. Biomed. Anal..

[40] Zhou Y., Abbas F., Wang Z., Yu Y., Yue Y., Li X., Yu R., Fan Y. (2021). HS–SPME–GC–MS and Electronic Nose Reveal Differences in the Volatile Profiles of *Hedychium* Flowers. Molecules.

[41] Zhou J., Feng T., Ye R. (2015). Differentiation of Eight Commercial Mushrooms by Electronic Nose and Gas Chromatography-Mass Spectrometry. J. Sens..

[42] Guo Q., Adelina N.M., Hu J., Zhang L., Zhao Y. (2022). Comparative Analysis of Volatile Profiles in Four Pine-Mushrooms Using HS-SPME/GC-MS and E-Nose. Food Control.

[43] Gholami R., Aghili Nategh N., Rabbani H. (2023). Evaluation the Effects of Temperature and Packaging Conditions on the Quality of Button Mushroom during Storage Using E-Nose System. J. Food Sci. Technol..

[44] Zhu M., Hu Z., Liang M., Song L., Wu W., Li R., Li Z., Zhang J. (2022). Evaluation of the Flavor Compounds of *Pleurotus eryngii* as Affected by Baking Temperatures Using HS-SPME-GC–MS and Electronic Nose. J. Food Process. Preserv..

[45] Chilo J., Pelegri-Sebastia J., Cupane M., Sogorb T. (2016). E-Nose Application to Food Industry Production. IEEE Instrum. Meas. Mag..

[46] Falasconi M., Concina I., Gobbi E., Sberveglieri V., Pulvirenti A., Sberveglieri G. (2012). Electronic Nose for Microbiological Quality Control of Food Products. Int. J. Electrochem..

[47] Mota I., Teixeira-Santos R., Cavaleiro Rufo J. (2021). Detection and Identification of Fungal Species by Electronic Nose Technology: A Systematic Review. Fungal Biol. Rev..

[48] Pei F., Yang W., Ma N., Fang Y., Zhao L., An X., Xin Z., Hu Q. (2016). Effect of the Two Drying Approaches on the Volatile Profiles of Button Mushroom (*Agaricus bisporus*) by Headspace GC–MS and Electronic Nose. LWT Food Sci. Technol..

[49] Chen D., Wang S., Li M., Hao T., Lin S. (2021). The Dynamic Changes in Product Attributes of Shiitake Mushroom Pilei and Stipes during Dehydration by Hot Air Drying. J. Food Process. Preserv..

[50] Ma N., Pei F., Yu J., Wang S., Ho C.T., Su K., Hu Q. (2018). Valid Evaluation of Volatile Flavor Composition of Fresh and Dehydrated *Tuber indicum* with Different Drying Methods. CyTA J. Food.

[51] Song Y., Hu Q., Wu Y., Pei F., Kimatu B.M., Su A., Yang W. (2019). Storage Time Assessment and Shelf-Life Prediction Models for Postharvest *Agaricus bisporus*. LWT.

[52] Portalo-Calero F., Arroyo P., Suárez J.I., Lozano J. (2019). Triangular Test of *Amanita* Mushrooms by Using Electronic Nose and Sensory Panel. Foods.

[53] Portalo-Calero F., Lozano J., Meléndez F., Arroyo P., Suárez J.I. (2019). Identification of Poisonous Mushrooms by Means of a Hand-Held Electronic Nose. Proceeding.

[54] Keshri G., Challen M., Elliott T., Magan N. (2003). Differentiation of *Agaricus* Species and Other Homobasidiomycetes Based on Volatile Production Patterns Using an Electronic Nose System. Mycol. Res..

[55] Gómez I., Lavega González R., Tejedor-Calvo E., Pérez Clavijo M., Carrasco J. (2022). Odor Profile of Four Cultivated and Freeze-Dried Edible Mushrooms by Using Sensory Panel, Electronic Nose and GC-MS. J. Fungi.

[56] Shi H., Zhang M., Adhikari B. (2017). Advances of Electronic Nose and Its Application in Fresh Foods: A Review. Crit. Rev. Food Sci. Nutr..

[57] AOAC (1990). AOAC Official Methods of Analysis.

[58] Mędyk M., Chudzińska M., Barałkiewicz D., Falandysz J. (2017). Specific Accumulation of Cadmium and Other Trace Elements in *Sarcodon imbricatus* Using ICP-MS with a Chemometric Approach. J Environ Sci Heal B.

[59] Medicine I. (2005). Dietary Reference Intakes for Energy, Carbohydrate, Fiber, Fat, Fatty Acids, Cholesterol, Protein, and Amino Acids.

[60] (2012). SCHER Assessment of the Tolerable Daily Intake of Barium.

[61] (2009). Scientific Opinion on Arsenic in Food. EFSA J..

[62] (2017). Dietary Reference Values for Nutrients Summary Report. EFSA Support. Publ..

[63] Turck D., Bohn T., Castenmiller J., de Henauw S., Hirsch-Ernst K.I., Knutsen H.K., Maciuk A., Mangelsdorf I., McArdle H.J., Peláez C. (2023). Scientific Opinion on the Tolerable Upper Intake Level for Selenium. EFSA J..

[64] Nutri-Score. https://www.santepubliquefrance.fr/en/nutri-score.

[65] Splivallo R., Ebeler S.E. (2015). Sulfur Volatiles of Microbial Origin Are Key Contributors to Human-Sensed Truffle Aroma. Appl. Microbiol. Biotechnol..

[66] Sensigents. https://www.sensigent.com/products/cyranose.html.

[67] Santos J.P., Lozano J.L., Aleixandre M., Sayago I., Fernández M.J., Arés L., Gutiérrez J., Horrillo M.D.C. (2004). Discrimination of Different Aromatic Compounds in Water, Ethanol and Wine with a Thin Film Sensor Array. Sens. Actuators B Chem..

[68] Murcia M.A., Martínez-Tomé M., Vera A., Morte A., Gutierrez A., Honrubia M., Jiménez A.M. (2003). Effect of Industrial Processing on Desert Truffles *Terfezia claveryi* Chatin and *Picoa juniperi* Vittadini): Proximate Composition and Fatty Acids. J. Sci. Food Agric..

[69] Jacinto-Azevedo B., Valderrama N., Henríquez K., Aranda M., Aqueveque P. (2021). Nutritional Value and Biological Properties of Chilean Wild and Commercial Edible Mushrooms. Food Chem..

[70] Roncero-Ramos I., Mendiola-Lanao M., Pérez-Clavijo M., Delgado-Andrade C. (2016). Effect of Different Cooking Methods on Nutritional Value and Antioxidant Activity of Cultivated Mushrooms. Int J Food Sci Nutr..

[71] Ekute B. (2019). Nutritional Profile of Two Nigerian Edible Mushrooms: *Pleurotus ostreatus* and *Pleurotus pulmonarius*. J. Appl. Sci. Environ. Manag..

[72] Yu Q., Guo M., Zhang B., Wu H., Zhang Y., Zhang L. (2020). Analysis of Nutritional Composition in 23 Kinds of Edible Fungi. J. Food Qual..

[73] Kalač P. (2013). A Review of Chemical Composition and Nutritional Value of Wild-Growing and Cultivated Mushrooms. J. Sci. Food Agric..

[74] Rybakowski J.K., Ferensztajn-Rochowiak E. (2022). Mini-Review: Anomalous Association between Lithium Data and Lithium Use. Neurosci. Lett..

[75] Siwulski M., Niedzielski P., Budka A., Budzyńska S., Kuczyńska-Kippen N., Kalač P., Sobieralski K., Mleczek M. (2022). Patterns of Changes in the Mineral Composition of *Agaricus Bisporus* Cultivated in Poland between 1977 and 2020. J. Food Compos. Anal..

[76] Siwulski M., Budka A., Budzyńska S., Gąsecka M., Kalač P., Niedzielski P., Mleczek M. (2021). Mineral Composition of Traditional and Organic-Cultivated Mushroom *Lentinula edodes* in Europe and Asia–Similar or Different?. LWT.

[77] Kalac P. (2019). Mineral Composition and Radioactivity of Edible Mushrooms.

[78] Manzi P., Gambelli L., Marconi S., Vivanti V., Pizzoferrato L. (1999). Nutrients in Edible Mushrooms: An Inter-Species Comparative Study. Food Chem..

[79] Royse D.J., Baars J., Tan Q. (2017). Current Overview of Mushroom Production in the World. Edible and Medicinal Mushrooms.

[80] Reyna S., Garcia-Barreda S. (2014). Black Truffle Cultivation: A Global Reality. For. Syst..

[81] WHO (2013). Global Action Plan for the Prevention and Control of Noncommunicable Diseases 2013–2020.

[82] van den Akker K., Bartelet D., Brouwer L., Luijpers S., Nap T., Havermans R. (2022). The Impact of the Nutri-Score on Food Choice: A Choice Experiment in a Dutch Supermarket. Appetite.

[83] Wang M., Zhao R. (2023). A Review on Nutritional Advantages of Edible Mushrooms and Its Industrialization Development Situation in Protein Meat Analogues. J. Future Foods.

[84] Kwasny T., Dobernig K., Riefler P. (2022). Towards Reduced Meat Consumption: A Systematic Literature Review of Intervention Effectiveness, 2001–2019. Appetite.

[85] Andreani G., Sogari G., Marti A., Froldi F., Dagevos H., Martini D. (2023). Plant-Based Meat Alternatives: Technological, Nutritional, Environmental, Market, and Social Challenges and Opportunities. Nutrients.

[86] Guinard J.X., Myrdal Miller A., Mills K., Wong T., Lee S.M., Sirimuangmoon C., Schaefer S.E., Drescher G. (2016). Consumer Acceptance of Dishes in Which Beef Has Been Partially Substituted with Mushrooms and Sodium has been Reduced. Appetite.

[87] Li J., Silver C., Gómez M.I., Milstein M., Sogari G. (2023). Factors Influencing Consumer Purchase Intent for Meat and Meat Substitutes. Future Foods.

[88] Lang M. (2020). Consumer Acceptance of Blending Plant-Based Ingredients into Traditional Meat-Based Foods: Evidence from the Meat-Mushroom Blend. Food Qual. Prefer..

[89] De Cianni R., Pippinato L., Mancuso T. (2023). A Systematic Review on Drivers Influencing Consumption of Edible Mushrooms and Innovative Mushroom-Containing Products. Appetite.

[90] Food Data Central. https://fdc.nal.usda.gov/fdc-app.html#/food-details/1999627/nutrients.

[91] Koutrotsios G., Danezis G., Georgiou C., Zervakis G.I. (2020). Elemental Content in Pleurotus Ostreatus and *Cyclocybe cylindracea* Mushrooms: Correlations with Concentrations in Cultivation Substrates and Effects on the Production Process. Molecules.

[92] Ahmad Zakil F., Xuan L.H., Zaman N., Alan N.I., Salahutheen N.A.A., Sueb M.S.M., Isha R. (2022). Growth Performance and Mineral Analysis of *Pleurotus Ostreatus* from Various Agricultural Wastes Mixed with Rubber Tree Sawdust in Malaysia. Bioresour. Technol. Rep..

[93] Hoa H.T., Wang C.L., Wang C.H. (2018). The Effects of Different Substrates on the Growth, Yield, and Nutritional Composition of Two Oyster Mushrooms (*Pleurotus ostreatus* and *Pleurotus cystidiosus*). Mycobiology.

[94] Shimokawa T., Kinoshita A., Kusumoto N., Nakano S., Nakamura N., Yamanaka T. (2020). Component Features, Odor-Active Volatiles, and Acute Oral Toxicity of Novel White-Colored Truffle *Tuber japonicum* Native to Japan. Food Sci. Nutr..

[95] Quintana-Rodriguez E., Rivera-Macias L.E., Adame-Alvarez R.M., Torres J.M., Heil M. (2018). Shared Weapons in Fungus-Fungus and Fungus-Plant Interactions? Volatile Organic Compounds of Plant or Fungal Origin Exert Direct Antifungal Activity In Vitro. Fungal Ecol..

[96] Combet E., Henderson J., Eastwood D.C., Burton K.S. (2006). Eight-Carbon Volatiles in Mushrooms and Fungi: Properties, Analysis, and Biosynthesis. Mycoscience.

[97] Maga J.A. (1981). Mushroom Flavor. J. Agric. Food Chem..

[98] Zhang H., Peng J., Zhang Y.R., Liu Q., Pan L.Q., Tu K. (2020). Discrimination of Volatiles of Shiitakes (*Lentinula Edodes*) Produced during Drying Process by Electronic Nose. Int. J. Food Eng..

[99] Feng T., Yang M., Ma B., Zhao Y., Zhuang H., Zhang J., Chen D. (2021). Volatile Profiles of Two Genotype Agaricus Bisporus Species at Different Growth Stages. Food Res. Int..

[100] Tagkouli D., Bekiaris G., Pantazi S., Anastasopoulou M.E., Koutrotsios G., Mallouchos A., Zervakis G.I., Kalogeropoulos N. (2021). Volatile Profiling of *Pleurotus eryngii* and *Pleurotus ostreatus* Cultivated on Agricultural and Agro-Industrial by-Products. Foods.

[101] Tasaki Y., Kobayashi D., Sato R., Hayashi S., Joh T. (2019). Variations in 1-Octen-3-Ol and Lipoxygenase Gene Expression in the Oyster Mushroom *Pleurotus Ostreatus* According to Fruiting Body Development, Tissue Specificity, Maturity, and Postharvest Storage. Mycoscience.

[102] Splivallo R., Ottonello S., Mello A., Karlovsky P. (2011). Truffle Volatiles: From Chemical Ecology to Aroma Biosynthesis. New Phytol..

[103] Sun L.B., Zhang Z.Y., Xin G., Sun B.X., Bao X.J., Wei Y.Y., Zhao X.M., Xu H.R. (2020). Advances in Umami Taste and Aroma of Edible Mushrooms. Trends Food Sci. Technol..

[104] Czapski G.A., Czubowicz K., Strosznajder R.P. (2012). Evaluation of the Antioxidative Properties of Lipoxygenase Inhibitors. Pharmacol. Rep..

[105] Nkadimeng S.M., Steinmann C.M.L., Eloff J.N. (2021). Anti-Inflammatory Effects of Four Psilocybin-Containing Magic Mushroom Water Extracts in Vitro on 15-Lipoxygenase Activity and on Lipopolysaccharide-Induced Cyclooxygenase-2 and Inflammatory Cytokines in Human U937 Macrophage Cells. J. Inflamm. Res..

[106] Mashima R., Okuyama T. (2015). The Role of Lipoxygenases in Pathophysiology; New Insights and Future Perspectives. Redox Biol..

[107] Szydłowska-Tutaj M., Szymanowska U., Tutaj K., Domagała D., Złotek U. (2023). The Addition of Reishi and Lion’s Mane Mushroom Powder to Pasta Influences the Content of Bioactive Compounds and the Antioxidant, Potential Anti-Inflammatory, and Anticancer Properties of Pasta. Antioxidants.

[108] Szydłowska-Tutaj M., Szymanowska U., Tutaj K., Domagała D., Złotek U. (2023). Influence of Addition of Dried Maitake and Enoki Mushrooms on Antioxidant, Potentially Anti-Inflammatory, and Anti-Cancer Properties of Enriched Pasta. Appl. Sci..

[109] Darwish R.S., Shawky E., Nassar K.M., Rashad ElSayed R.M., Hussein D.E., Ghareeb D.A., El Sohafy S.M. (2021). Differential Anti-Inflammatory Biomarkers of the Desert Truffles *Terfezia claveryi* and *Tirmania nivea* Revealed via UPLC-QqQ-MS-Based Metabolomics Combined to Chemometrics. LWT.

[110] Choo K.S.O., Bollen M., Dykes G.A., Coorey R. (2021). Aroma-Volatile Profile and Its Changes in Australian Grown Black Périgord Truffle (*Tuber melanosporum*) during Storage. Int. J. Food Sci. Technol..

[111] Mohd Ali M., Hashim N., Abd Aziz S., Lasekan O. (2020). Principles and Recent Advances in Electronic Nose for Quality Inspection of Agricultural and Food Products. Trends Food Sci. Technol..

