# Supplementary material for: Using Science and Technology to Unveil The Hidden Delicacy Terfezia arenaria, a Desert Truffle
Source: Foods. 2023 Sep 22;12(19):3527. doi: 10.3390/foods12193527 (PMC10572273; doi:10.3390/foods12193527)
Supplement: Supplementary file 1 [file foods-12-03527-s001.zip › foods-2583438-supplementary.pdf]

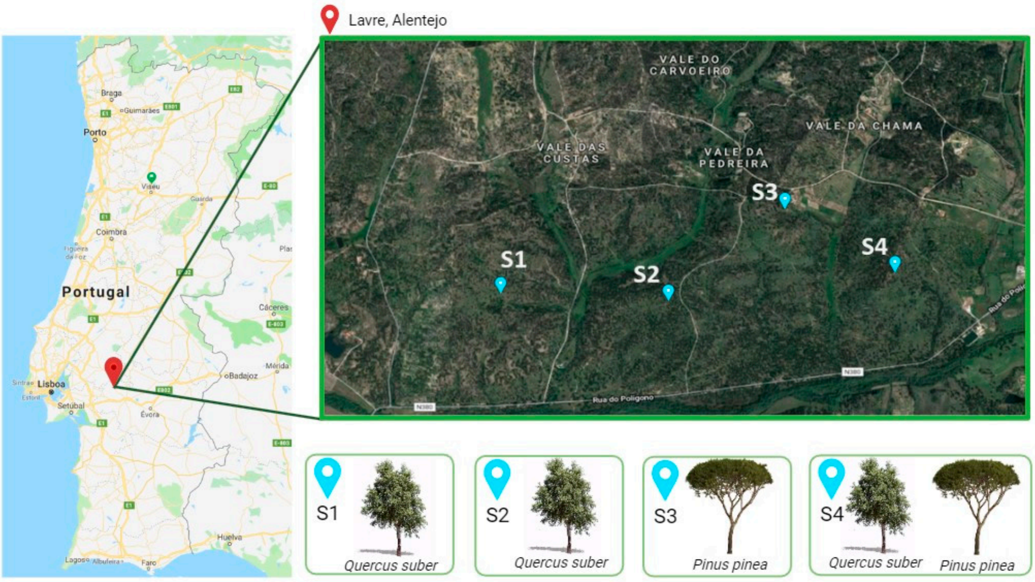

Figure S1 – Map of the sampling areas of *Tefezia arenaria* collected during this study and forest-dominant species per area.

Table S1 - Parameters settings for using the Cyranose-320.

Method setting	Parameter setting	Pump speed
Baseline purge	10 sec	Medium
Sample draw	10 sec	Medium
Air intake purge	5 sec	High
Sample gas purge	30 sec	High
Digital filtering	On	
Substrate heater	On: 42°C	
Training repeat count 1	1	
Identifying repeat count 1	1	
Statistical analysis by PCnose		
Algorithm	Canonical	
Pre-processing	Auto-scaling	
Normalization	Normalization 1	
Identification Quality	Medium	

Table S2 – Dietary Reference Intakes for nutrients and elements. This table presents the Recommended Dietary Allowances (RDA, shown in bold), Adequate Intakes (AI, identified with *) and Tolerable Upper Intake Level (UL, identified with †). These values were adapted from Dietary Reference Intakes Datasets from the USA, [61], and the EU [62–65]. The two right-most columns show the contribution (in %) of an 100 g intake of dry and fresh *T. arenaria*, to each nutrient and mineral element, considering the RDA or AI values.

			Adults (>18)			*Terfezia arenaria*	
		**Unit**	**Males**	**Females**	**UL**	**100 g dry**	**100 g fresh**
Nutrients	Carbohydrates	g/day	130	130		55 %	< 1 %
	Total Fiber	g/day	30 - 38*	21 - 25*		48 %	< 1 %
	Protein [a]	g/day	56	46		33 %	< 1 %
	Fat	g/day	ND	ND			
Elements	Cr	(µg/day)	30 - 35*	20 - 25*	ND	> 100%	3 % - 6 %
	Li	(µg/day)	ND	ND	2 [b,d]	> 100%	2 %
	Se	(µg/day)	55	55	255	91%	1 %
	Cu	(µg/day)	900	900	10,000	73%	< 1 %
	P	(mg/day)	700	700	4000	20 %	< 1 %
	Fe	(mg/day)	8	8 - 18	45	11 % - 24 %	< 1 %
	K	(mg/day)	3400*	2600*	ND	10 % - 14 %	< 1 %
	Zn	(mg/day)	11	8	40	10% - 13 %	< 1 %
	Mn	(mg/day)	2.3*	1.8*	11	6 % - 11 %	< 1 %
	Mg	(mg/day)	420	310 - 320	350	3 % - 4 %	< 1 %
	As†	(µg/kg bw per day)	ND	ND	< 15	1 %[b]	0.01% [c]
	Ba†	(mg/kg bw per day)	ND	ND	0.2	< 1 % [b]	< 0.01% [c]
	Ca	(mg/day)	1000 - 1200	1000 - 1200	2500	< 1 %	< 0.01%
	Mo	(µg/day)	45	45	2000	< 1 %	< 0.01%
	Na	(mg/day)	1500*	1500*	2300	< 1 %	< 0.01%
	Ni	(mg/day)	ND	ND	1.0	< 1 % [c]	< 0.01%[d]

[a] Based on g of protein per kg of body weight for the reference body weight, e.g., for adults 0.8 g kg^{-1} of body weight for the reference body weight.
[b] Provisional reference dose.
[c] Values considering a person with 60 kg of reference body weight.
[d] Was considered the UL value.
† Elements with detrimental health effects
ND: Not determined.

Table S3 – Comparison of the abundance of the main VOCs identified in *Terfezia arenaria* and in the other edible mushroom and truffle species. Values for *T. arenaria* were determined in the present study while values for the other mushrooms and truffle were determined in other studies. Abundance: +++ high; ++ medium; + low; - absent.

**Compounds**		***Terfezia arenaria***	***Agaricus bisporus***	***Lentinula edodes***	***Pleurotus ostreatus***	***Tuber melanosporum***
Alcohols	1-Octen-3-ol	+++	+++	+++	++	+
	3-Octanol	+	++	+	++	+
	2-Octen-1-ol	+	+	+	-	-
Aldehydes	Benzeneacetaldehyde	+	+	+	-	+
	Hexanal	+	-	-	+	+
	2-Octenal	+	+	+	+	-
	Nonanal	+	-	-	+	-
Hydrocarbons	Tetradecane	+	+	-	-	-
Ketones	3-Octanone	++	++	++	++	+
Terpenes	Limonene	+	+	-	+	-
**References**		This study	[99]	[98]	[100]	[110]

Table S4 –Characterization of the VOCs identified in *Terfezia arenaria*, and in the other edible mushroom and truffle species (*Agaricus bisporus*, *Lentinula edodes*, *Pleurotus ostreatus* and *Tuber melanosporum*). The quantity of each volatile is presented in % of the total VOCs detected. The results for *Terfezia arenaria* were originated from this study, while the other mushroom species data was collected from literature review (Continue).

	**Compounds**	***Terfezia arenaria***	***Agaricus bisporus***	***Lentinula edodes***	***Pleurotus ostreatus***	***Tuber melanosporum***
Alcohols	1-Octen-3-ol	64.411%	35.919%	35.909%	28.437%	0.226%
	3-Octanol	1.734%	23.040%	5.813%	27.538%	0.192%
	1-Octanol		1.369%	4.139%	0.789%	0.011%
	Phenylethyl Alcohol		0.036%	0.341%		0.802%
	3-methyl-1-Butanol					17.877%
	1-Hexanol				0.507%	0.011%
	2-ethyl-1-hexanol				0.140%	0.011%
	2-butanol					0.440%
	1-Propanol, 2-methyl					3.173%
	1-Butanol					0.011%
	1-Butanol, 2-methyl-					25.793%
	1-Pentanol					0.011%
	1-Butanol, 2-ethyl					0.011%
	1-Pentanol, 4-methyl-					0.011%
	2-Heptanol, 6-methyl-					0.011%
	1-Hexanol, 3-methyl					0.011%
	Ethanol, 2-(methylthio)-					0.090%
	trans-(2-Ethylcyclopentyl)methanol					0.011%
	1-Propanol, 3-(methylthio)-					0.113%
	(Z)-2-Octen-1-ol	3.625%	1.102%	3.187%		
	Benzyl Alcohol		1.920%	0.170%		
	Benzeneethanol, β-methyl-			0.271%		
	1-Nonanol		0.124%			

Table S4 – Continue.

	**Compounds**	*Terfezia arenaria*	*Agaricus bisporus*	*Lentinula edodes*	*Pleurotus ostreatus*	*Tuber melanosporum*
Alcohols	3-methyl-1-Butanol		0.071%			
	3-Nonanol		0.053%			
	3-Heptanol		0.036%			
	(5Z)-Octa-1,5-dien-3-ol	1.554%				
	Chlorohexanol	0.158%				
	1-Dodecanol	0.113%				
Sulphurs	Disulfide, dimethyl			0.882%		0.056%
	Dimethylsulfide					9.972%
	Carbon disulfide			8.118%		
	Lenthionine			5.332%		
	Dimethyl trisulfide			4.630%		
	Tetrasulfide, dimethyl			1.303%		
	1,2,4-Trithiolane			1.042%		
	1,2,4,5-Tetrathiane			0.291%		
	Cyclic octaatomic sulfur			0.702%		
Acids	Acetic acid		0.107%	0.431%		0.056%
	Propanoic acid, 2-methyl-					0.068%
	Butanoic acid,4-hydroxy-					0.011%
	Butanoic acid, 2-methyl-					1.299%
	Cystine			0.551%		
	n-Hexadecanoic acid			0.170%		
	Pentadecanoic acid			0.080%		
	Tetradecanoic acid			0.261%		
	Propanoic acid		0.053%			

Table S4 – Continue.

	**Compounds**	*Terfezia arenaria*	*Agaricus bisporus*	*Lentinula edodes*	*Pleurotus ostreatus*	*Tuber melanosporum*
Aldehydes	Benzaldehyde		5.566%	0.822%	0.300%	0.045%
	Benzeneacetaldehyde	0.236%	0.249%	0.401%		0.011%
	Hexanal	4.830%			1.438%	0.011%
	Octanal			0.261%	0.407%	0.011%
	2-Methyl-Butanal		0.036%			0.316%
	3-Methyl-Butanal				0.860%	0.474%
	Acetaldehyde					3.320%
	Butanal					0.011%
	2-Butenal					0.440%
	4-Methyl-Hexanal					0.011%
	5-Methyl-Hexanal					0.011%
	(E)-2-Octenal	0.968%	2.009%	0.601%	1.240%	
	Nonanal	0.135%			0.032%	
	trans-2-hexenal / (E)-2-Hexenal				0.127%	
	2,4-nonadienal				0.144%	
	2-Phenylpropenal			9.421%		
	2-Phenylpropionaldehyde			0.231%		
	2-Propenal, 3-phenyl-			0.261%		
	(E, E)-2,4-Octadienal		0.302%			
	Benzaldehyde, 2,5-bis[(trimethylsilyl)oxy]	0.214%				
Hydrocarbons	2,4-Dithiapentane					0.023%
	Benzene, 1-methoxy-3-methyl-					8.357%
	Benzene, 1,2-dimethoxy-					0.440%

Table S4 – Continue.

	**Compounds**	*Terfezia arenaria*	*Agaricus bisporus*	*Lentinula edodes*	*Pleurotus ostreatus*	*Tuber melanosporum*
Hydrocarbons	Benzene, 1,3-dimethoxy-					0.011%
	Benzene, 1,4-dimethoxy-2-methyl-					0.124%
	Butane, 1-methoxy-2-methyl-					0.124%
	Anisole					6.098%
	Toluene				7.548%	1.016%
	Undecane		0.018%		1.414%	
	Dodecane		0.036%	0.040%	0.305%	
	Nonadecane				0.160%	
	Pentadecane			0.100%	0.478%	
	Heptadecane			0.210%	0.319%	
	Hexadecane	0.169%	0.018%	0.140%	0.393%	
	cis-α-Bisabolene		0.036%		0.360%	
	Octadecane				0.262%	
	Eicosane	0.090%			0.107%	
	2-Methyl-2-phenyl-Oxirane			1.012%		
	2-Methyl-2-phenyl-Oxirane		0.036%			
	Decane		0.036%			
	Tetradecane	0.236%	0.231%			
	Dotriacontane	0.315%				
	Eicosane-7-hexyl	0.236%				
	3,3,5-Trimethylheptane	0.146%				
	1-chloroeicosane	0.113%				
	Caprylene (1-octene)	0.293%				

Table S4 – Continue.

	Compounds	*Terfezia arenaria*	*Agaricus bisporus*	*Lentinula edodes*	*Pleurotus ostreatus*	*Tuber melanosporum*
Ketones	3-Octanone	14.479%	19.027%	11.736%	25.880%	0.203%
	2-Butanone					3.817%
	2-Pentanone					0.384%
	4-Heptanone					0.011%
	Acetone		0.213%			1.863%
	2-Hexanone, 5-methyl-					0.011%
	2-Heptanone, 6-methyl-					0.011%
	Acetoin					0.113%
	2,3-octanedione				0.101%	
	2-Undecanone		0.036%	0.080%		
	1-Isoindolinone			0.932%		
	2-Octanone		0.302%			
	(E)-6,10-dimethyl-5,9-Undecadien-2-one		0.551%			
	1-Octen-3-one		2.792%			
	3-Nonanone		0.036%			
	3-Cyclohepten-1-one		0.925%			
	2-Octanone, 1-nitro-	0.259%				
	Geranylacetone	0.101%				
Esters	Hexadecanoic acid ethyl ester		0.071%			
	Hexanedioic acid, bis(2-ethylhexyl) ester		3.539%			
	Formic acid,1-methylethyl ester					0.011%
	1-Butanol, 2-methyl-, acetate					0.395%
	Ethyl acetate					0.113%

Table S4 – Continue.

	**Compounds**	*Terfezia arenaria*	*Agaricus bisporus*	*Lentinula edodes*	*Pleurotus ostreatus*	*Tuber melanosporum*
Esters	Ethane, 1,1-diethoxy-					1.852%
	Propanoic acid, 2-methyl-, ethyl ester					0.011%
	Butanoic acid, 2-methyl-ethyl ester					2.428%
	Butanoic acid, 3-methyl-ethyl-ester					0.429%
	Formic acid, 2-methylbutylester					0.892%
	Propanoic acid, 2-methyl-, 2-methylpropyl ester					0.011%
	Propanoic acid ,2-methyl-, 2-methylbutyl ester					0.011%
	Butyl 2-methylbutanoate					0.011%
	Butanoic acid, 2-methyl-, 2-methylbutyl ester					1.739%
	Butanoic acid, 3-methyl-, 2-methylbutyl ester					0.011%
	Pentanoic acid, 2-methylbutyl ester					0.192%
	Methyl palmitate			0.130%		
	Pentyl propanoate	1.103%				
	Propionic acid, 3-iodo-, octadecyl ester	0.439%				
	Propanoic acid, 2-methyl-, 3-hydroxy-2,2,4-trimethylpentyl ester	0.248%				
Terpenes	Pristane				0.360%	
	Phytane				0.328%	
	Limonene	0.394%	0.107%		0.025%	
	α-Pinene	2.105%				

Table S4 – Characterization of the VOCs identified in *Terfezia arenaria*, and in the other edible mushroom and truffle species (*Agaricus bisporus*, *Lentinula edodes*, *Pleurotus ostreatus* and *Tuber melanosporum*).

	**Compounds**	*Terfezia arenaria*	*Agaricus bisporus*	*Lentinula edodes*	*Pleurotus ostreatus*	*Tuber melanosporum*
Other compounds	Carbon dioxide					4.337%
	1-Octadecanesulphonyl chloride	0.338%				
	Anthranilic acid	0.146%				
	Tyrosol	0.124%				
	Henicosanoic acid	0.327%				
	Pyridine, 5-ethenyl-2-methyl-	0.360%				
References		This Study	[99]	[98]	[100]	[110]

Table S5 – Results and rates of Cyranose-320 identification of *T. arenaria*, *A. bisporus*, *L. edodes*, *P. ostreatus* and *T. melanosporum* with 40 °C and RT pre-analysis incubation temperatures.

	Pre-analysis 40 °C			Pre-analysis RT		
**Sample**	**Result**	**Rate[a]**		**Result**	**Rate[a]**	
Terfezia arenaria	Terf3	*****	excellent	Terf3	*****	excellent
Terfezia arenaria	Terf3	*****	excellent	Terf2, Terf2		
Terfezia arenaria	Terf3	*****	excellent	Terf3	*****	excellent
Terfezia arenaria	Terf1	*****	excellent	Terf3	***	acceptable
Terfezia arenaria	Terf3	*	not acceptable	Terf3	*	not acceptable
Terfezia arenaria	Terf3, Terf3			Terf2, Terf2		
Terfezia arenaria	Terf2	*	not acceptable	Terf3	*****	excellent
Terfezia arenaria	Terf2	*	not acceptable	Terf3	*****	excellent
Terfezia arenaria	Terf2, Terf2			Terf3	***	acceptable
Terfezia arenaria	Terf3	*	not acceptable	Terf3	*****	excellent
Terfezia arenaria	Terf2	*	not acceptable	Terf2	*****	excellent
Terfezia arenaria	Terf2	*****	excellent	Terf3	*****	excellent
Terfezia arenaria	Terf2	*****	excellent	Terf3	*****	excellent
Terfezia arenaria	Terf2	*****	excellent	Terf3	***	acceptable
Terfezia arenaria	Terf2	*****	excellent	Terf3	*****	excellent
Terfezia arenaria	Terf3	***	acceptable	Terf3	***	acceptable
Terfezia arenaria	Terf2, Terf3			Terf3	*****	excellent
Terfezia arenaria	Terf3	*	not acceptable	Terf3	*	not acceptable
Terfezia arenaria	Terf3	*	not acceptable	Terf3	*****	excellent
Terfezia arenaria	Terf3, Terf3			Terf3	*****	excellent
Agaricus bisporus	Terf2	*****	excellent	Unknown		
Agaricus bisporus	Terf3	*	not acceptable	Confused		
Agaricus bisporus	Terf1	*****	excellent	Confused		
Agaricus bisporus	Terf1	***	acceptable	Terf1	***	acceptable
Agaricus bisporus	Terf2	*	not acceptable	Terf1	*****	excellent
Lentinula edodes	Confused			Terf3	***	acceptable
Lentinula edodes	Terf2	*****	excellent	Unknown		
Lentinula edodes	Terf1	*****	excellent	Unknown		
Lentinula edodes	Terf1	*****	excellent	Unknown		
Lentinula edodes	Confused			Terf3	*****	excellent
Pleurotus ostreatus	Confused			Unknown		
Pleurotus ostreatus	Confused			Terf3	***	acceptable
Pleurotus ostreatus	Terf1	***	acceptable	Terf3	*	not acceptable
Pleurotus ostreatus	Confused			Unknown		
Pleurotus ostreatus	Confused			Unknown		
Tuber melanosporum	Unknown			Unknown		
Tuber melanosporum	Unknown			Terf1	***	acceptable
Tuber melanosporum	Unknown			Terf2	*	not acceptable
Tuber melanosporum	Unknown			Unknown		
Tuber melanosporum	Unknown			Unknown		

[a] Result rate with stars, where: 5 stars (*****) - 100% of probability – excellent
4 stars (****) - 80% of probability - good
3 stars (***) - 60% of probability – acceptable
2 stars (**) - 40% of probability – bad
1 star (*) - 20% of probability – not acceptable
